# Europium(III) Meets Etidronic Acid (HEDP): A Coordination Study Combining Spectroscopic, Spectrometric, and Quantum Chemical Methods

**DOI:** 10.3390/molecules28114469

**Published:** 2023-05-31

**Authors:** Anne Heller, Christian Senwitz, Harald Foerstendorf, Satoru Tsushima, Linus Holtmann, Björn Drobot, Jerome Kretzschmar

**Affiliations:** 1 Chair of Radiochemistry/Radioecology, Faculty of Chemistry and Food Chemistry, Technische Universität Dresden, 01062 Dresden, Germany; christian.senwitz1@tu-dresden.de; 2 Central Radionuclide Laboratory, Radiation Protection Office, Technische Universität Dresden, 01062 Dresden, Germany; 3 Institute of Resource Ecology, Helmholtz-Zentrum Dresden-Rossendorf, 01328 Dresden, Germany; h.foerstendorf@hzdr.de (H.F.); s.tsushima@hzdr.de (S.T.); b.drobot@hzdr.de (B.D.); 4 International Research Frontiers Initiative (IRFI), Institute of Innovative Research, Tokyo Institute of Technology, Tokyo 152-8550, Japan; 5 Institute of Radioecology and Radiation Protection, Leibniz Universität Hannover, 30419 Hannover, Germany; holtmann@irs.uni-hannover.de

**Keywords:** lanthanides, TRLFS, NMR, ATR-FT-IR, DFT, ESI-MS, complexation, speciation

## Abstract

Etidronic acid (1-Hydroxyethylidene-1,1-diphosphonic acid, HEDP, H_4_L) is a proposed decorporation agent for U(VI). This paper studied its complex formation with Eu(III), an inactive analog of trivalent actinides, over a wide pH range, at varying metal-to-ligand ratios (M:L) and total concentrations. Combining spectroscopic, spectrometric, and quantum chemical methods, five distinct Eu(III)−HEDP complexes were found, four of which were characterized. The readily soluble EuH_2_L^+^ and Eu(H_2_L)_2_^−^ species with log β values of 23.7 ± 0.1 and 45.1 ± 0.9 are formed at acidic pH. At near-neutral pH, EuHL^0^_s_ forms with a log β of ~23.6 and, additionally, a most probably polynuclear complex. The readily dissolved EuL^−^ species with a log β of ~11.2 is formed at alkaline pH. A six-membered chelate ring is the key motif in all solution structures. The equilibrium between the Eu(III)–HEDP species is influenced by several parameters, i.e., pH, M:L, total Eu(III) and HEDP concentrations, and time. Overall, the present work sheds light on the very complex speciation in the HEDP–Eu(III) system and indicates that, for risk assessment of potential decorporation scenarios, side reactions of HEDP with trivalent actinides and lanthanides should also be taken into account.

## 1. Introduction

In case of accidental incorporation, heavy metals, particularly radionuclides, pose a serious health risk to humans. To minimize the risk and to enhance the excretion of incorporated heavy metal noxa, decorporation agents binding the respective metal ion or radionuclide with high selectivity are needed. In the case of tri- and tetravalent actinides and lanthanides, the commonly used drug is diethylenetriaminepentaacetic acid (DTPA) [1]. However, DTPA is nearly ineffective in uranium(VI) decorporation due to the formation of weak U(VI)–DTPA complexes. In contrast, etidronic acid shows a high affinity for U(VI) and, therefore, was proposed as a suitable decorporation agent for U(VI) [1,2,3,4]. However, since it is more likely that not only one specific nuclide but rather a mixture of various (interrelated) elements (radionuclide vector) is incorporated, investigations concerning the suitability of etidronic acid to sequester other radionuclides such as trivalent actinides are of crucial importance.

**Figure 1:**
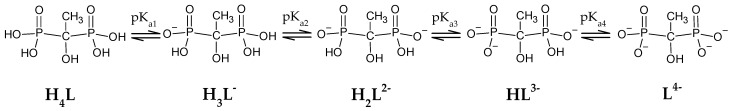
Generic structures of HEDP’s protonation species together with the abbreviations used throughout this work.

Etidronic acid is a diphosphonic acid (1-Hydroxyethylidene-1,1-diphosphonic acid) which is diversely used, e.g., as a chelating agent as well as antiscalant and corrosion inhibitor in industry and technology [5,6,7], carrier of radiopharmaceuticals [8,9], and as a pharmaceutical for the treatment of osteoporosis and bone atrophy [10,11]. The versatile use is based on the polydentate chemical structure, containing two phosphonate groups and one hydroxyl group. This is also why etidronic acid has many other names and abbreviations, e.g., HEDPA, EHBP, EHDP, EDPA, and HEDP. In this paper, for the sake of simplicity, we use the abbreviation HEDP. Generic structures of the several protonation species are given in Figure 1.

Owing to its wide use, the properties of HEDP are well-studied, and p*K*_a_ values were reported for various ionic strengths and media (see Supplementary Materials, Table S1). However, data for physiological ionic strength and media *I* = 0.1 M (NaCl) are scarce but mandatory for HEDP with regard to in vivo applications.

Concerning the complexation of metals with HEDP, there are plenty of studies for alkali and transition metals, e.g., refs. [12,13,14,15,16,17]. However, few data is available for lanthanides commonly used as non-radioactive analogs for radioactive trivalent actinides [18,19,20,21,22,23,24,25,26,27]. Especially coordination compounds of lanthanides with HEDP prepared by hydrothermal syntheses, ranging from 120 to 230 °C for up to seven days and often in water-methanol mixtures, are reported along with their solid-state structures established by single-crystal X-ray diffraction (e.g., refs. [22,25,27,28,29]). These structures reveal seven- or eight-coordinated lanthanide ions with H_2_L^2−^ and/or HL^3−^ ligand(s) chelating one or chelating and bridging between two metal ions and a six-membered ring as the binding motif [22,25,27,28,29]. In contrast, data on the formation of aqueous HEDP complexes with lanthanides are scarce [19,20,21,23] (see also IUPAC technical report [30]). At pH 1–3, and millimolar concentrations, the simultaneous formation of five different Eu(III)–HEDP complexes, along with their stability constants, is reported by ref. [23] using solvent extraction. At similar acidic pH, a complex with the H_2_L^2−^ ligand species and a proposed structure is reported for several lanthanides [19,20].

In the present study, the fundamental interactions of HEDP with Eu(III) were studied over the whole range of 0.5 < pH < 13 and 10^−6^ < [Eu] < 10^−3^ M using complementary spectroscopic, spectrometric, and quantum chemical methods. Particular attention was paid to the speciation of aqueous Eu(III)–HEDP complexes and the elucidation of the actual solution structure of each species. Eu(III) was chosen as a suitable analog for trivalent actinides due to its well-known chemical similarities for many inorganic and organic (bio-) ligands, e.g., refs. [31,32,33,34]. Furthermore, Eu(III) exhibits unique luminescence properties enabling complex formation and speciation studies even at trace metal concentrations using time-resolved laser-induced fluorescence spectroscopy (TRLFS). The solubility in the Eu(III)–HEDP system was investigated with mass spectroscopy using inductively coupled plasma (ICP-MS). Structural details on the formed complex species were obtained by combining nuclear magnetic resonance (NMR) and infrared (IR) spectroscopy with mass spectrometry using electrospray ionization (ESI-MS) and quantum chemical calculation using density functional theory (DFT). Three parameters were studied regarding their influence on the complex formation: (i) total metal and ligand concentrations (both 10^−6^–10^−3^ M), (ii) metal to ligand ratio (M:L; 1:0.1–1:10), and (iii) pH value (pH = 0.5–13). Furthermore, regarding the potential use of HEDP as a decorporation agent and preceding cell culture experiments, the formation of Eu(III)–HEDP complexes in the cell culture medium was studied. The presented data provide a comprehensive image of this complex ligand system and the various species formed with Eu(III) and presumably trivalent actinides in an aqueous solution.

## 2. Results and Discussion

The results are presented according to the information needed for HEDP’s intended use as a decorporation agent: (i) p*K*_a_ values of HEDP for physiological ionic strength and media, (ii) the solubility of Eu(III) as a non-radioactive analog of trivalent actinides in the presence of HEDP, (iii) structural information on the Eu(III)–HEDP species formed, (iv) thermodynamic characterization of the fundamental Eu(III)–HEDP system, and (v) interaction of Eu(III) and HEDP in cell culture medium.

### 2.1. HEDP Characterization and pK_a_ Values at I = 0.1 M (NaCl)

The pure ligand was characterized over the 0.5 < pH < 13 range using NMR, IR spectroscopy, and DFT. The pH-dependent ^1^H and ^31^P NMR spectra are given as Supplementary Materials, Figures S1 and S2. The p*K*_a_ values in Table 1 were derived from the spectra of both nuclei. In general, our p*K*_a_ values agree with most of the literature data (see Supplementary Materials, Table S1) [13,14,15,16,23,30,35,36,37]. However, the results of refs. [38,39] could not be verified since the p*K*_a_ value of the hydroxyl group must be in the order of >14, comparable to other alcoholic hydroxyl groups. This appears to be plausible because the release of a proton from a small, already fourfold negatively charged molecule is expected to occur only under harsh conditions in aqueous media. Ref. [40] reported this p*K*_a_ to be >14.6. Nevertheless, in the presence of appropriate Lewis acids, deprotonation was observed at pH values below the corresponding p*K*_a_ value [41]. Consequently, such behavior was considered for the subsequent metal ion complexation studies. Using our p*K*_a_ values at *I* = 0.1 M (NaCl), the aqueous speciation of HEDP was calculated and is provided in Supplementary Materials, Figure S3.

The IR spectra of HEDP measured within the range of 0.5 < pH < 13 and those of the distinct HEDP species are provided as Supplementary Materials, Figures S4 and S5A. A tentative assignment of the vibrational bands is given in Supplementary Materials, Table S2. In general, the spectra are in good agreement with published literature [42,43,44]. However, our measurements provide additional spectral data within an extended pH range.

The pattern of the vibrational bands representing the phosphonate groups allows the identification of different protonation species prevailing under the respective conditions. In general, the spectra of species showing a higher molecule symmetry, i.e., H_2_L^2−^ and L^4−^, are dominated by two strong bands representing the ν(P=O) and ν(POO) modes, whereas with decreasing the molecule’s symmetry (H_3_L^−^ and HL^3−^) an increased number of bands is observed mainly due to the splitting of the ν(POO) modes. These observations were considered for the subsequent metal ion complexation studies.

In addition to the experimental data, the IR spectra of the HEDP species were obtained from quantum-chemical calculations using DFT (see Supplementary Materials, Figure S5B). The theoretical IR spectra agree with the experimental data and widely reproduce the spectral changes with increasing pH. Furthermore, the DFT calculations revealed intramolecular solid hydrogen bonds within the HEDP molecule.

In summary, NMR and IR spectroscopy consistently demonstrate the presence of five HEDP species in aqueous solution according to the different protonation states. Furthermore, the respective p*K*_a_ values were calculated, and a distinct pH range of (nearly) exclusive existence was found for each species. The obtained spectra serve as a reference for interpreting the spectra recorded in Eu(III) containing HEDP solutions.

### 2.2. Solubility of Europium(III) in the Presence of HEDP

**Figure 2:**
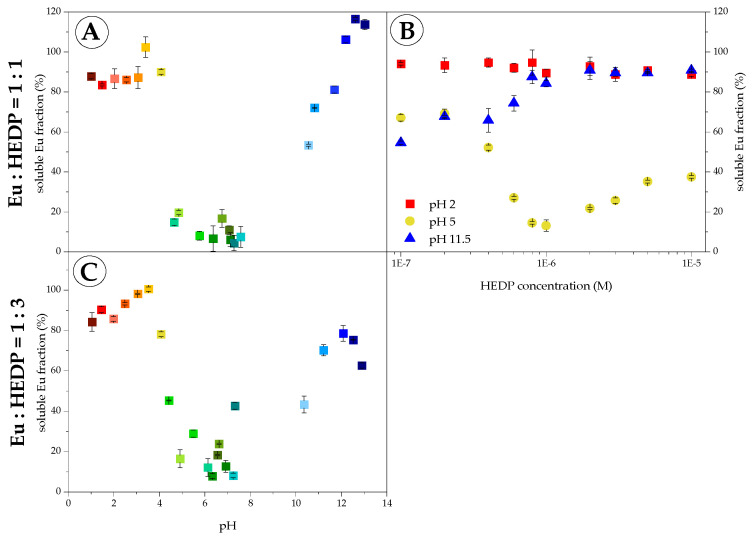
The soluble fraction in the Eu(III)–HEDP system as determined by ICP-MS in supernatants of TRLFS pH-titration series (**A**,**C**) and HEDP concentration series at constant pH 2, 5, and 11.5 (**B**) at 10^−6^ M Eu(III), without background electrolyte and at room temperature (colors are based on the pH from acidic = red to alkaline = blue).

The solubility in the Eu(III)–HEDP system was studied using ICP–MS after (ultra-) centrifugation. Eu(III) concentrations have been determined in suspensions and supernatants of HEDP concentration series at constant pH 2, 5, and 11.5 as well as of pH-titration series at constant M:L of both 1:1 and 1:3. Various Eu(III) concentrations, viz. 10^−6^ and 10^−5^ M (TRLFS) as well as 10^−3^ M (IR, NMR) were applied. Figure 2 and Table S3, Supplementary Materials present the solubility in the Eu(III)–HEDP system at micromolar concentrations.

In the case of the pH-titration series (Figure 2A,C), no significant differences in solubility were observed for both applied M:L. Within the limits of ICP-MS, the total initial amount of Eu(III) is recovered in the supernatants at acidic pH 1–4. In contrast, between pH 4 and 8, the solubility curve exhibits a sharp drop, and less than 20% of the initial Eu(III) concentration is recovered in the supernatants. This proves that a hardly soluble Eu(III)–HEDP species is formed and precipitates. At alkaline pH 10–12, Eu(III) solubility re-increases and >50% of the initial Eu(III) content is recovered in the supernatants. These findings are verified by solubility results of the HEDP concentration series at constant pH (Figure 2B). At pH 2, Eu(III) is completely soluble up to tenfold ligand excess, whereas at pH 5 Eu(III) solubility is reduced to 60% even at tenfold HEDP deficit and further decreases with increasing ligand concentration. This, again, demonstrates the precipitation of a hardly soluble Eu(III)–HEDP complex. As for ligand excess, increased solubility indicates a competing process. At pH 11.5, Eu(III) solubility is low for HEDP deficit because of strong Eu(III) hydrolysis. Upon increasing ligand concentration, Eu(III) solubility increases and reaches 90% when HEDP is in excess, proving a successful outcompetition of Eu(III) hydrolysis by the formation of a soluble Eu(III)–HEDP species.

At millimolar concentrations, precipitation occurred in the pH range from ~2 to 11, indicating a preferred formation of hardly soluble Eu(III)–HEDP species at higher metal concentrations. The solubility curves of the corresponding pH-titration series and the ICP-MS data are provided as Supplementary Materials, Table S4 and Figure S6. Furthermore, precipitates varied in color and appearance depending on pH and M:L, indicating the formation of several precipitating complexes, presumably those species hereafter referred to as *complexes 3* and *4* (vide infra). At first glance, in some samples, the precipitate appeared as a white powder, whereas in others, it was colorless and jelly-like, presumably due to polymerization. However, a detailed characterization of the precipitates is beyond the scope of this paper and is the subject of future investigations.

In summary, Eu(III) solubility in the presence of HEDP is both pH- and concentration-dependent. At acidic and alkaline pH, Eu(III) solubility is high, indicating soluble Eu(III)–HEDP species, whereas it is low at near-neutral pH and hardly soluble Eu(III)–HEDP species precipitate. At higher Eu(III) concentrations, this precipitation occurs fast and for a wider pH range.

### 2.3. Structural Investigations on Soluble Europium(III)–HEDP Complexes

To elucidate the structure of Eu(III)–HEDP species, different methods were applied. The stoichiometry of complexes was determined using ESI-MS. The functional groups of HEDP involved in the Eu(III) complexation and the binding mode were determined with NMR and IR spectroscopy.

In the case of ESI-MS, measurements were possible only at pH 2 due to enhanced viscosity of samples at near-neutral pH, presumably resulting from the formation of hardly soluble complex species and/or polymerization. Experiments were performed at 10^−4^ M Eu(III) with increasing HEDP concentration but without background electrolyte to prevent strong signal clusters arising from NaCl. According to HEDP’s speciation in the acidic range, the following complexes with H_3_L^−^ and H_2_L^2−^ were considered: EuH_3_L^2+^, Eu(H_3_L)_2_^+^, EuH_2_L^+^, and Eu(H_2_L)_2_^−^. The results from ESI-MS are provided as Supplementary Materials, Figure S7 and Table S5. In positive mode, the signals from the Eu^3+^ aqua ion dominated for ligand deficit and were detectable up to fivefold ligand excess. Eu(III)–HEDP complexes with 1:1 stoichiometry were detected over the whole HEDP concentration range reaching their maximum as the predominant fraction for nearly equimolar M:L. Eu(III)–HEDP complexes with 1:2 stoichiometry emerge as ofequimolar M:L and increase in concentration upon HEDP excess. In negative mode, no peaks were detected at ligand deficit, whereas, at ligand excess, significant peaks of a 1:2 complex appear. An unambiguous assignment of the complex species measured is impossible due to the inherent methodical problem of adduct formation. Nevertheless, in summary, ESI-MS results prove semi-quantitatively the formation of at least one Eu(III)–HEDP complex with 1:1 stoichiometry at acidic pH over the whole HEDP concentration range investigated (M:L between tenfold ligand deficit and excess) as well as at least one species with 1:2 stoichiometry existing for HEDP excess.

IR spectroscopic measurements of the aqueous Eu(III)–HEDP species were feasible only at very acidic and alkaline pH values due to the low solubility of complexes at millimolar concentrations. All experimental spectra obtained at pH 0.5 and 12.5 and two different M:L ratios (i.e., equimolar and threefold ligand excess) are provided as Supplementary Materials, Figure S8. Under highly acidic conditions, the spectra proved the coordination of the Eu^3+^ ion to the ligand. According to the reference spectra of the H_3_L^−^ and H_2_L^2−^ species (cf. Figure S5A), the appearance of the ν_as_(POO) mode at 1079 cm^−1^ in the spectrum of the Eu-complex as well as the simultaneous reduction of the intensities of the δ(POH) mode at 1015 cm^−1^ and to a lower extent of the coupled ν_as+s_(POOH) modes at 928 cm^−1^, respectively, demonstrate the release of a proton from HEDP upon complexation of Eu(III). These spectral changes became more obvious in the corresponding difference spectrum (Supplementary Materials, Figure S8A, third and fifth traces from top). In particular, this spectrum revealed a broad positive band in the spectral range of the ν(P=O) mode around 1150 cm^−1^ most likely reflecting a change of the electrostatic conditions of this functional group upon coordination of the Eu cation. It has to be noted that the spectra of the Eu(III)–HEDP complexes still showed contributions from the fully protonated H_4_L ligand, indicating free HEDP still being present in the solution.

At pH 12.5, the IR spectra again proved the coordination of the Eu^3+^ ion to the phosphonate functional groups. The absorption, as well as the difference spectra, revealed significant shifts to higher frequencies of the ν(P=O) and ν_as_(POO) modes of about 30 cm^−1^ and of the ν_s_(POO) mode of about 40 cm^−1^, respectively. The spectra recorded at threefold ligand excess showed less pronounced spectral changes than the pure ligand spectrum, indicating an increased fraction of noncomplexed HEDP under these conditions. However, the very good agreement of the difference spectra at both M:L demonstrated that the same Eu(III)–HEDP complexes are formed (Supplementary Materials, Figure S8B, third and fifth traces from top).

The derivation of the Eu-binding mode in the HEDP complexes from the IR spectra is challenging. Nevertheless, a contribution of the ligand’s hydroxyl group to the Eu(III) coordination appears unlikely since a release of the proton from this functional group is expected to generate a characteristic ν(C–O) mode of high intensity, as it was previously observed by ref. [32]. Moreover, the doubly degenerated stretching mode (ν_3_) of conjugated triatomic functional groups and its spectral splitting (referred to as ν_as_ and ν_s_) due to coordination to metal ions might be taken as a figure of merit for the binding mode [45,46]. In general, an increased and decreased spectral splitting (Δν_3_) represents monodentate and bidentate coordination, respectively. However, the extent of Δν_3_ observed in the spectra of this work appeared equivocally and may be interpreted in two ways resulting in increased or decreased values for Δν_3_ (see Figure S8B). Since in the spectra no redshift of the ν_as_(POO) mode was observed (what would be necessary for an assignment to a bidendate binding), it is suggested that the coordination of HEDP to Eu(III) occurs in the same binding mode as in the reference spectrum of the ligand where Na^+^ serves as a counterion, which is most likely monodentate coordinated.

For NMR spectroscopic measurements in the Eu(III)–HEDP system, the same solubility limitations as for IR spectroscopy apply. All spectra recorded at pH 0.5 and 12.5 are provided as Supplementary Materials, Figures S9 and S10. Since, owing to fast ligand exchange reactions, the observed signals are molar fraction-weighted averages of free and Eu(III)-bound HEDP, interpretation of the spectra obtained at pH 0.5 is straightforward. Upon increasing Eu(III) concentration, both the ^1^H and the ^31^P NMR signals successively shift upfield as they become increasingly Eu(III)–HEDP complex-weighted. Remarkably, the ^31^P signal shifts upfield by over (−)60 ppm, proving the formation of a soluble Eu(III)–HEDP complex at a pH as low as 0.5.

The results from the NMR spectra at pH 12.5 are somewhat different and more complex. In the case of ligand excess, regardless of the M:L, the spectra are very similar, and the emerging signals are inconspicuous, merely indicating some interaction between Eu(III) and HEDP. For equimolar M:L, however, ^1^H and ^31^P NMR signals remarkable in both intensity and chemical shift appear in addition to the minor ones observable at ligand excess. Noteworthy, these prominent signals are all of comparable intensity. Considering the high concentrations applied for IR and NMR spectroscopies, the formation of polynuclear species is very likely. The fourteen distinct ^31^P signals imply that this complex species comprises seven HEDP ligand molecules, most likely fourfold anionic at pH 12.5, thus assuming that the polynuclear species is highly negatively charged and hence of high solubility. The structure of this complex species is subject to further investigation.

In summary, several indications on the structure of the individual Eu(III)–HEDP species formed at very acidic and alkaline pH, i.e., the stoichiometry, the protonation state of the ligand, and the binding mode, were derived from ESI-MS as well as NMR and IR spectroscopy.

### 2.4. Thermodynamic Studies of the Fundamental Europium(III)–HEDP System

The thermodynamics of the Eu(III)–HEDP system was studied using TRLFS. To enable complexation with different ligand species, Eu(III) spectra were recorded at constant pH with increasing HEDP concentration (i.e., varying M:L) as well as at constant Eu(III) and HEDP concentrations (i.e., constant M:L) and varying pH. All experiments were performed at 10^−6^ and 10^−5^ M Eu(III).

The formation of Eu(III)−HEDP species was observed at each pH. The TRLF spectra of the several complexes differ by shape and intensity of the emission bands (^5^D_0_ → ^7^F_j_, j = 0, …, 4) and the luminescence lifetime in dependence on the HEDP concentration and the pH. TRLF spectra of HEDP concentration series at constant pH values are provided as Supplementary Materials, Figures S11 and S12. However, comprehensive data evaluation of such a complex system can only be accomplished with the help of mathematical tools. Therefore, the parallel factor analysis (PARAFAC) results are discussed in the following.

**Figure 3:**
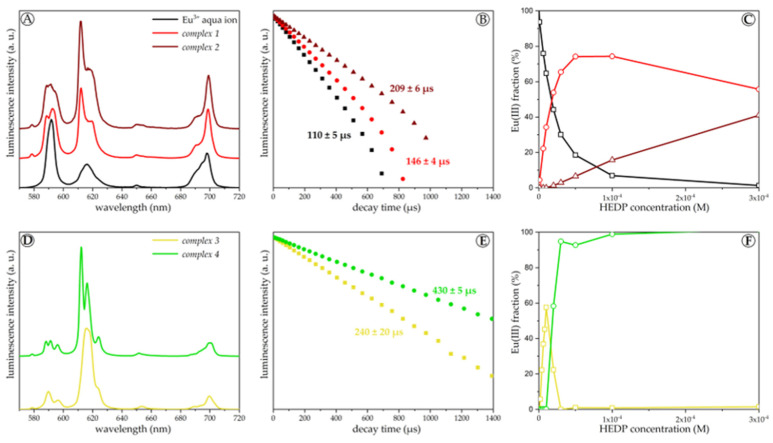
Steady-state luminescence spectra (**A**,**D**), luminescence decay curves (**B**,**E**) and speciation (**C**,**F**) of 10^−5^ M Eu(III) at pH 2 (**A**–**C**) and pH 5 (**D**–**F**), *I* = 0.1 M (NaCl) and (25 ± 1) °C in dependence on the HEDP concentration (steady-state spectra are normalized to the ^7^F_1_ band area, and decay curves are normalized to the luminescence intensity at *t* = 0).

In the acidic region at pH 2, an increasing HEDP concentration leads to the formation of two different consecutive Eu(III) complexes (*complexes 1* and *2*, Figure 3 top). Their emission spectra reveal an increased R_E/M_ (ratio of the ^7^F_2_ band over the ^7^F_1_ band), the appearance of a small ^7^F_0_ band, and prolonged luminescence lifetimes. Overall, the shape of the TRLF spectra of both Eu(III)−HEDP complexes exhibit similar features to those of other phosphate-dominated Eu(III) binding motifs, e.g., in nucleic acids [47].

In the near-neutral region at pH 5, again, an increasing HEDP concentration leads to the formation of two consecutive Eu(III) complexes (*complexes 3* and *4*, Figure 3 bottom) accompanied by turbidity of the solution. This reflects the solubility issue described in Section 2.2. Due to the phase separation, these hardly soluble Eu(III)−HEDP species are assigned to net neutral complexes. The emission spectra of both species are characterized by a small ^7^F_0_ peak and a high R_E/M_. Furthermore, the ^7^F_1_ and ^7^F_2_ bands are significantly split, and the luminescence lifetimes are long.

**Figure 4:**
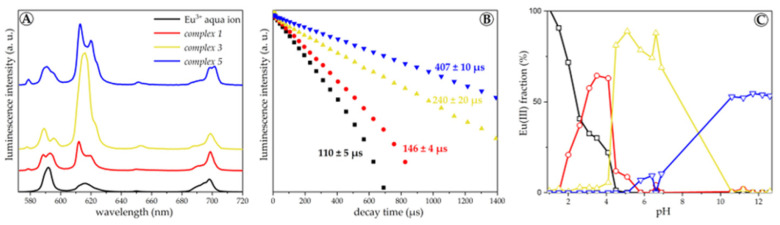
Steady-state luminescence spectra (**A**), luminescence decay curves (**B**) and speciation (**C**) of 10^−6^ M Eu(III) at equimolar M:L, without background electrolyte and at (25 ± 1) °C in dependence on the pH (steady-state spectra are normalized to the ^7^F_1_ band area, and decay curves are normalized to the luminescence intensity at *t* = 0).

In the alkaline region at pH 11.5, with increasing ligand concentration, the HEDP complexation competes with Eu(III) hydrolysis and an Eu(III)−HEDP complex distinct from the other ones is formed (*complex 5*, Figure 4). As a result, its emission spectrum exhibits a small ^7^F_0_ band, a high R_E/M_, and a long luminescence lifetime. In addition, the splitting of the ^7^F_4_ band is enhanced.

These results were verified by additional series with constant M:L and varying pH. The respective TRLF spectra and luminescence decay curves are provided as Supplementary Materials, Figures S13 and S14. At equimolar M:L and acidic pH, *complex 1* dominates (see Figure 4). Upon increasing the pH to ~4, the speciation changes, *complex 3* is formed, and the solution turns turbid. However, when the pH is raised further and exceeds ~9, the precipitate dissolves, and the solution turns clear again, corresponding to the formation of *complex 5*. At ligand excess, the speciation differs in the near-neutral pH range by the additional formation of *complex 4*. With increasing Eu(III) concentration, this complex species predominates.

Based on the emission spectra, all five Eu(III)−HEDP complex species are distinguishable but share the same general features. The appearance of the small ^7^F_0_ band indicates a slight distortion in the symmetry of the metal center of all complexes. An increase in the R_E/M_ is usually associated with the strength of the ligand field. Hence, the hardly soluble *complexes 3* and *4* should have the strongest ligand field of all five species. The luminescence lifetime corresponds to the displacement of water molecules from the first hydration shell of Eu(III) by the ligand [48]. With increasing pH, more water molecules are replaced by HEDP and in *complexes 4* and *5*, only two water molecules are left. The luminescence spectroscopic parameters of all complexes characterized are given in Supplementary Materials, Table S6.

**Figure 5:**
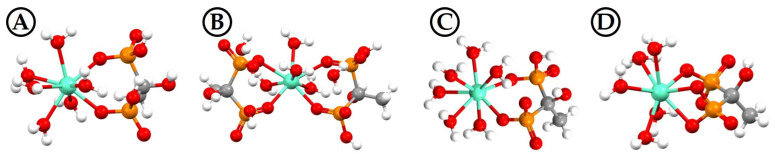
DFT calculated solution structures of the EuH_2_L^+^ (**A**), Eu(H_2_L)_2_^−^ (**B**), EuHL^0^_s_ (**C**), and EuL^−^ (**D**) complexes.

To identify the solution structure of the individual Eu(III)−HEDP species, different model structures were calculated with DFT. Based on the results from all experimental studies, the following species were assumed: EuH_2_L^+^ as *complex* 1, Eu(H_2_L)_2_^−^ as *complex 2*, EuHL^0^_s_ as *complex 3*, and EuL^−^ as *complex 5*. For *complex 4*, experimental results are insufficient to deduce an unambiguous complex structure. However, combining the results obtained so far, *complex 4* presumably is a species with HL^3−^ (or NaL^3−^) coordinating to and bridging between Eu(III), finally forming a polymeric net-neutral and, hence, hardly soluble precipitate. For each Eu(III)–HEDP species, structure models with different binding modes were calculated, i.e., monodentate, bidentate, and chelate binding of Eu(III) via one or both phosphonate groups of the HEDP ligand. All structure models considered are provided in Supplementary Materials, Figure S15, and the finally assigned solution structure of each Eu(III)–HEDP complex is given in Figure 5.

For each complex species, the energetically most stable structure model is a chelate involving at least one six-membered ring between Eu(III) and both phosphonate groups. This matches the monodentate binding fashion deduced from IR spectra (Eu(III) binding via one oxygen of each phosphonate group). It is well known that such chelate rings significantly stabilize complex structures and render them energetically favorable over bidentate structures. Therefore, most structures reported for HEDP–metal ion complexes involve this six-membered ring motif [2,14,17,20,22,24,49]. In the case of EuH_2_L^+^ (Figure 5A), almost the same solution structure was reported for the analogous NdH_2_L^+^ complex [20]. The coordination number of Eu(III) is reduced from nine for the Eu^3+^ aqua ion to eight in all four complexes.

Although solid-state structures may not necessarily represent those found in the solution-state, especially when obtained under notably different conditions (e.g., room temperature and an aqueous solution of defined pH value vs. hydrothermal syntheses), at least recurring binding motifs can be identified and validated. Single-crystal structures of Ln(III)–HEDP coordination compounds reveal seven- or eight-coordinated Ln(III) ions with HEDP chelating one or chelating and bridging between two metal ions, whereby the ligand species are predominantly H_2_L^2−^ and/or HL^3−^, respectively, and the binding motif is a six-membered ring [22,25,27,28,29]. Considering the above-discussed arguments and that neither of the hydrothermal syntheses applied alkaline conditions, the four DFT-calculated and spectroscopically underpinned solution structures are reasonable.

With a known solution structure, the species distribution within the TRLFS series can be used to determine complex formation constants. This was done using PARAFAC as shown elsewhere [50,51], and log *K* and log β values were calculated according to the equations in Table 2. Computational details are given in Supplementary Materials.

Literature data on aqueous lanthanide–HEDP complexes is sparse and only exists for pH < 3. However, our complex formation constants for EuH_2_L^+^ and EuHL^0^_s_ agree with data from refs. [19,20]. Differences in the data reported by refs. [22,23] can be mainly attributed to the different ionic strengths and techniques used for constant determination.

In summary, using TRLFS, five distinct Eu(III)–HEDP species have been identified in dependence on the pH and the M:L. All species were characterized, and for four of them, we could eventually address the actual solution structures and calculate the respective complex formation constants.

### 2.5. Europium(III) Complex Formation with HEDP in Cell Culture Medium

To investigate the stability of the Eu(III)–HEDP complex(es) under biological conditions, the complex formation was studied in cell culture medium at both equimolar M:L and threefold ligand excess in dependence on the total Eu(III) concentration (10^−5^ and 10^−3^ M), incubation time (1 and 24 h), and the order of adding the single constituents, i.e., (i) Eu(III) + HEDP mixed first, then added to the medium (EH), (ii) first adding Eu(III) to the medium, then HEDP (ME), and (iii) first adding HEDP to the medium, then Eu(III) (MH). Luminescence spectra were recorded using TRLFS, and the fraction of soluble Eu(III) was determined by ICP-MS.

Results of all measurements are provided in Supplementary Materials, Figure S17. At micromolar concentrations, only 40–70% of Eu(III) is soluble in EH samples, whereas in ME and MH samples Eu(III) is completely soluble. At millimolar concentrations and equimolar M:L, Eu(III) quantitatively precipitates in EH samples independent from incubation time. Solubility is higher in ME and MH samples but significantly reduced to 10–30%. Concerning the incubation time, the data show that Eu(III) solubility is decreasing with incubation time, indicating slow kinetics accompanied by proceeding precipitation of the Eu(III)–HEDP species formed. Comparing data of samples with the same order of addition but varying M:L reveals that Eu(III) solubility is significantly lower at equimolar M:L than at ligand excess. This might indicate the formation of Eu(III)–HEDP species with different solubility. In general, solubility results demonstrate that all parameters investigated influence the Eu(III) complex formation in cell culture and that components of the medium and HEDP compete for Eu(III) complexation.

For all ICP-MS samples, TRLF spectra of suspensions and supernatants were measured. In some cases, precipitates were visible after centrifugation and measured separately. At both 10^−5^ and 10^−3^ M Eu(III), the spectra are quite similar for all ME and MH samples. The ^7^F_1_ and ^7^F_2_ bands are broad, and the luminescence lifetime is very long (τ = 800–1000 µs). In contrast, at 10^−5^ M Eu(III), precipitation occurred in almost all EH samples and TRLF spectra of the precipitates exhibit the following significant spectral alterations compared to that of the corresponding supernatants: (i) both the ^7^F_1_ and ^7^F_2_ bands are split and exhibit a distinct fine structure and (ii) the luminescence lifetime is significantly shorter (τ = 450–550 µs). For EH samples at millimolar concentration and equimolar M:L, the same observations were made. However, no precipitate was observed at ligand excess, and the TRLF spectrum in the supernatant is similar to that of ME and MH samples. These results indicate two distinct species with different solubility: (i) a hardly soluble Eu(III)–HEDP complex dominating in EH samples, particularly at equimolar M:L, and (ii) a soluble Eu(III) species with HEDP and/or medium constituent(s) dominating in ME and MH samples. In all cases, no significant spectral differences were observed between samples incubated for 1 and 24 h.

Comparing the spectra of Eu(III) and HEDP in a cell culture medium with those of the several Eu(III)–HEDP complexes and that of only Eu(III) in a cell culture medium enables the identification of the two Eu(III) species. Data are provided as Supplementary Materials, Figure S18. The steady-state spectra and luminescence lifetimes measured in ME and MH samples are very similar to that of the Eu(III) complex(es) formed with (a) protein(s) of fetal bovine serum (FBS) from cell culture medium [52,53]. In contrast, the TRLF spectra of precipitates from EH samples exhibit common features with that of *complex 4* (EuHL^0^_s_), i.e., particularly the fine structure of the split ^7^F_2_ band and the shorter luminescence lifetime compared to the Eu(III)–protein complex(es). This demonstrates the competition of HEDP and proteins from FBS for the Eu(III) complexation.

In summary, ICP-MS and TRLFS results are in good agreement, and both demonstrate that the M:L, total concentrations applied, and incubation time all influence the solubility of Eu(III) and HEDP in the cell culture medium. Mixing Eu(III) and HEDP before adding to the cell culture medium leads to the formation of hardly soluble EuHL^0^_s_. Proteins from FBS can only partially compete with HEDP and re-solubilize Eu(III) into the cell culture medium, particularly at micromolar concentrations and HEDP excess, respectively. In contrast, when mixing Eu(III) or HEDP with the cell culture medium before the addition of the ligand or metal, respectively, seems to favor the formation of (a) soluble Eu(III)–protein species. HEDP can partially compete with the protein(s) but only at higher Eu(III) and HEDP concentrations. At micromolar concentrations, the Eu(III)–protein species remains stable and in solution. However, the competition of HEDP and protein(s) is time-dependent, and with prolonged incubation time, the equilibrium is more shifted towards HEDP, resulting in decreased Eu(III) solubility.

## 3. Conclusions

For the first time, the complexation of Eu(III) with HEDP was comprehensively investigated over the entire pH range at varying M:L and total concentrations using multiple complementary spectroscopic and spectrometric techniques and quantum chemical calculations. As a result, five distinct Eu(III)–HEDP complexes were characterized by TRLFS, four of which were characterized in detail with NMR and IR spectroscopy as well as ICP- and ESI-MS, along with DFT eventually solving their molecular structure and, finally, with the help of PARAFAC, determining their respective formation constants.

We demonstrated that, on the one hand, all these complexes can be discriminated concerning their luminescence spectroscopic features but, on the other hand, exact structural characterization is complicated by at least two obstacles: (i) the simultaneous formation of several complexes in solution and (ii) the very low solubility of EuHL^0^_s_ and *complex 4* combined with their predominance at near-neutral pH.

Furthermore, the complex equilibrium between the several Eu(III)–HEDP species is influenced by several parameters, with the most important one being the pH determining the protonation state of HEDP in a certain complex. Second, the M:L determines the stoichiometry of the Eu(III)–HEDP species: 1:1 complexes are favored at ligand deficit up to equimolar M:L, whereas 1:2 complexes prevail at ligand excess. Third, the total Eu(III) and HEDP concentrations influence the formation of hardly soluble complexes and their pH range of existence. Finally, time also becomes essential when formation kinetics are slow, as is the case for the hardly soluble Eu(III)–HEDP complexes.

The interactions of Eu(III) and HEDP were also studied in a cell culture medium at physiological pH. We demonstrated that the most important parameter influencing this system is the order of mixing the individual components. Mixing Eu(III) and HEDP first leads to significantly lower solubility, i.e., a higher fraction of Eu(III)–HEDP species, than mixing cell culture medium with Eu or HEDP first. Corresponding to refs. [52,53], the soluble Eu(III) fraction was identified as (a) Eu(III) complex(es) with protein(s) from FBS. In contrast, the hardly soluble fraction very much resembles *complex 4*. This proves a competition of proteins from the cell culture medium and HEDP for Eu(III) binding.

Furthermore, with increasing incubation time, the Eu(III) solubility is decreasing, i.e., kinetics are slow, but in the end, the thermodynamically favored *complex 4* outcompetes the Eu(III)–protein species. Concerning preceding cell culture studies and HEDP’s potential application as a decorporation agent, these results raise some concerns about its applicability. Consequently, for risk assessment of potential decorporation scenarios, side reactions of HEDP with trivalent actinides and lanthanides should also be considered.

Overall, the present work sheds light on the complex interactions between Eu(III) and HEDP, provides unprecedented and valuable formation constants for thermodynamic databases as well as spectroscopic data, and solution structures of four distinct complex species. Further investigations aiming at the structure of both yet unknown Eu(III)–HEDP species, i.e., *complex 4* and the polynuclear species occurring at very alkaline pH, millimolar Eu(III) concentrations, and equimolar M:L, are in progress.

## 4. Materials and Methods

Experiments covered aqueous sample series at varying pH, Eu(III) total concentration, M:L, and were studied by TRLFS, IR and NMR spectroscopy, ESI-MS, and ICP-MS. All preparations and measurements were performed under an ambient atmosphere at (25 ± 1) °C. Experiments were complemented by quantum chemical calculations at the DFT level. Comprehensive experimental and computational details are provided as Supplementary Materials.

## Figures and Tables

**Table 1:** Acid dissociation constants of HEDP were obtained from NMR pH-titration data. Values represent averages determined from both ^1^H and ^31^P NMR spectra.

Site	p*K*_a_ ± SD (*I* = 0.1 M NaCl)	p*K*_a_^0^ (*I* → 0) ^1^
-PO_3_H_2_	1.36 ± 0.35	1.57
-PO_3_H_2_	2.46 ± 0.19	2.89
-PO_3_H^−^	6.93 ± 0.14	7.57
-PO_3_H^−^	11.12 ± 0.09	11.98
-OH	>14	>15

^1^ Values are extrapolated to *I* = 0 using the Davies Equation.

**Table 2:** Formation constants of Eu(III)–HEDP complexes at *I* = 0 M determined in this study.

Equation	Eu:H:L	log *K*^0^	log β^0^	Literature Data
Eu^3+^ + H_2_L^2−^ ⇌ EuH_2_L^+^	1:2:1	5.6 ± 0.4	–	5.81 ± 0.05 [19] ^1^ 4.6 ± 0.2 (I = 2 M) [23] ^2^ 5.62 ± 0.07 (Nd(III)) [20] ^1^
Eu^3+^ + 2 H_2_L^2−^⇌ EuH_4_L_2_^−^	1:4:2	10.5 ± 0.2	–	–
Eu^3+^ + HL^3−^ ⇌ EuHL^0^_s_	1:1:1	15.2 ± 0.2	–	6.4 ± 0.2 (I = 2 M) [23] ^2^
Eu^3+^ + L^4−^ ⇌ EuL^−^	1:0:1	13.8 ± 0.2	13.8 ± 0.2	–
Eu^3+^ + L^4−^ + H^+^ ⇌ EuHL^0^_s_	1:1:1	–	27.2 ± 0.2	19.9 (I = 2 M) [22]
Eu^3+^ + 2 L^4−^ + 4 H^+^ ⇌ EuH_4_L_2_^−^	1:4:2	–	49.6 ± 0.2	–
Eu^3+^ + L^4−^ + 2 H^+^ ⇌ EuH_2_L^+^	1:2:1	–	25.2 ± 0.4	–

^1^ Data obtained from spectrographic titration, ionic strength not reported. ^2^ Data obtained from liquid-liquid extraction at pH 1–3, evaluated by and taken from ref. [30].

## Data Availability

All data are included in this paper.

## Supplementary Materials

The following supporting information can be downloaded at: https://www.mdpi.com/article/10.3390/molecules28114469/s1, Materials and Methods (detailed); Characterization of the HEDP ligand (detailed), Figures S1 and S2: NMR spectra and chemical shift values of HEDP in dependence on pH, Figure S3: Speciation diagram of pure HEDP in aqueous solution, Figure S4: IR spectra of HEDP in dependence on the pH, Figure S5: experimental and DFT-calculated IR spectra of single HEDP species, Table S1: Literature data of p*K*_a_ values for HEDP, Table S2: Tentative assignment of the IR absorption bands of HEDP; Solubility of europium(III) in the presence of HEDP (detailed), Figure S6: Solubility in the Eu(III)–HEDP system as determined by ICP-MS, Tables S3 and S4: Solubility in the Eu(III)–HEDP system; Structural investigations on soluble europium(III)–HEDP complexes (detailed), Figure S7: Stoichiometry and distribution of Eu(III)–HEDP complexes at pH 2 as determined by ESI-MS, Table S5: ESI-MS data of the Eu(III)–HEDP system at pH 2, Figure S8: IR spectra of the Eu(III)–HEDP system at pH 0.5 and 12.5, Figure S9: NMR spectra of the Eu(III)–HEDP system at pH 0.5, Figure S10: NMR spectra of the Eu(III)–HEDP system at pH 12.5; Thermodynamic studies of the europium(III)−HEDP system (detailed), Figures S11 and S12: TRLFS spectra of the Eu(III)–HEDP system at 10^−6^ and 10^−5^ M in dependence on the M:L, Figures S13 and S14: TRLFS spectra of the Eu(III)–HEDP system at 10^−6^ and 10^−5^ M in dependence on the pH, Table S6: Luminescence spectroscopic properties of the Eu(III) species determined in this study, Figure S15: DFT-calculated structure models taken into account, Figure S16: Eu(III) hydrolysis constants reported in literature; Europium(III) complex formation with HEDP in cell culture medium (detailed), Figure S17: Solubility in the Eu(III)–HEDP–cell culture medium system as determined by ICP-MS, Figure S18: TRLFS spectra of the Eu(III)–HEDP–cell culture medium system. References [13,14,15,16,23,32,33,35,36,37,38,39,40,48,50,54,55,56,57,58,59,60,61,62,63,64,65,66,67,68,69,70,71,72,73,74,75,76,77,78,79,80,81,82,83,84,85,86] are cited in the Supplementary Materials.

## Sample Availability

Samples of the compounds are not available from the authors.
