# Supplementary material for: Europium(III) Meets Etidronic Acid (HEDP): A Coordination Study Combining Spectroscopic, Spectrometric, and Quantum Chemical Methods
Source: Molecules. 2023 May 31;28(11):4469. doi: 10.3390/molecules28114469 (PMC10254137; doi:10.3390/molecules28114469)
Supplement: Supplementary file 1 [file molecules-28-04469-s001.zip › molecules-2415227-supplementary.pdf]

Europium(III) Meets Etidronic Acid (HEDP): a Coordination Study Combining Spectroscopic, Spectrometric, and Quantum Chemical Methods

Anne Heller[1,2,*], Christian Senwitz[1,2], Harald Foerstendorf[3], Satoru Tsushima[3,4], Linus Holtmann[5], Björn Drobot[3], and Jerome Kretzschmar[3,*]

[1] Chair of Radiochemistry/Radioecology, Faculty of Chemistry and Food Chemistry, Technische Universität Dresden, 01062 Dresden, Germany; christian.senwitz1@tu-dresden.de (C. S.)
[2] Central Radionuclide Laboratory, Radiation Protection Office, Technische Universität Dresden, 01062 Dresden, Germany
[3] Institute of Resource Ecology, Helmholtz-Zentrum Dresden-Rossendorf, 01328 Dresden, Germany; h.foerstendorf@hzdr.de (H. F.); s.tsushima@hzdr.de (S. T.); b.drobot@hzdr.de (B. D.)
[4] International Research Frontiers Initiative (IRFI), Institute of Innovative Research, Tokyo Institute of Technology, Tokyo 152-8550, Japan
[5] Institute of Radioecology and Radiation Protection, Leibniz Universität Hannover, 30419 Hannover, Germany; holtmann@irs.uni-hannover.de (L. H.)
* Correspondence: anne.heller@tu-dresden.de (A. H.); j.kretzschmar@hzdr.de (J. K.)

Contents:

- Materials and Methods (detailed)
- Characterization of the HEDP ligand (detailed)
- Solubility of europium(III) in the presence of HEDP (detailed)
- Structural investigations on soluble europium(III)–HEDP complexes (detailed)
- Thermodynamic studies of the fundamental europium(III)–HEDP system (detailed)
- Europium(III) complex formation with HEDP in cell culture medium (detailed)

The references cited in this Supplementary Materials are listed in the reference list in the manuscript.

Materials and Methods (detailed)

Solutions and reagents

HEDP (1-hydroxyethan-(1,1-diphosphonic acid) was purchased as etidronic acid monohydrate ($C_2H_8O_7P_2 \cdot H_2O$, ≥ 95 %) from Sigma. $EuCl_3 \cdot 6\ H_2O$ (99.9 %) was purchased from Sigma, NaCl (extra

pure, ≥ 99.5 %) from Thermo Fisher Scientific, HCl, NaOH and KOH (each Reag. Ph. Eur.) from Roth. All chemicals were used without further purification. Stock solutions of each 0.1 M HEDP and Eu(III) were prepared by dissolving the respective amount in Milli-Q water. A stock solution of 1 M NaCl was used as background electrolyte to maintain constant ionic strength in the experiments. For comparison, some experiments were performed without any background electrolyte, denoted $I = 0$ M.

The pH values of the solutions were measured using a pH meter (pH 7110 from ino-Lab) equipped with a micro combination electrode (BlueLine16 pH from SI Analytics) and corrected with HCl and NaOH or KOH (for experiments with pH ≥ 10 in order to avoid alkali error at high pH values).

Sample preparation

In general, all sample preparation was carried out by dilution of stock solutions with Milli Q water or threefold distilled water under ambient atmosphere at (25 ± 1) °C. Due to kinetic processes in the Eu HEDP system, samples were prepared several days prior to the experiments (equilibration time 5 – 7 days).

For NMR, pure ligand samples with final concentrations of 10^{-3} M HEDP and 0.1 M background electrolyte were prepared. For samples with pH < 7 NaCl was used, for samples with pH ≥ 7 KCl in order to avoid alkali error at high pH values using a glass electrode (3 M KCl). The applicability of the two background electrolytes was validated with sample duplicates of the same pH (6.5 – 7.5) and ionic strength (0.1 M) but varying background electrolyte (NaCl and KCl), and can be seen from data points featuring smooth curves free of discontinuities. For determination of pK_a constants, the deprotonation of the pure HEDP ligand was studied from pH 0.52 to 12.75, with a step size of ~ 0.25 pH units. Hence, the titration series comprised 49 samples.

All ATR-FT-IR samples of the pure ligand were prepared with a final concentration of 5×10^{-3} M HEDP and an ionic strength of 0.1 M NaCl at seven pH values between pH 1 and 12. For investigating the complexation with Eu, samples with fixed HEDP concentration and metal to ligand ratios (M:L) of 1:1 and 1:3 were prepared at the same ionic strength and identical pH. Additionally, reference samples with only 0.1 M NaCl were prepared at the same pH values.

For TRLFS, two sets of samples were prepared. In samples of the first set, the Eu(III) concentration was 10^{-5} M, whereas in those of the second set, it was magnitude lower 10^{-6} M. Within both sets, ionic strength was kept constant at 0.1 M NaCl and sample series with analog parameters were prepared: i) concentration-titration series at constant pH 2, 5, 9, and 12 with M:L varying from 10:1 to 1:10 and ii) pH-titration series at constant M:L of 1:1 and 1:3 with varying pH from 0.5 to 13.0. Additionally, all samples of pH titration series were also prepared without constant ionic strength.

Samples for ESI-MS were prepared at constant pH 1, 2, 5, and 12 with 10^{-4} M Eu and varying M:L of 10:1 to 1:10. All samples were prepared without background electrolyte because, at a concentration of 0.1 M, NaCl leads to clogging of the needle.

Nuclear magnetic resonance spectroscopy (NMR)

NMR spectroscopy was performed using an Agilent DD2-600 NMR system, operating at 14.1 T with corresponding ^{1}H and ^{31}P resonance frequencies of 599.8 and 242.8 MHz, respectively, using a 5 mm oneNMR™ probe. For the measurements, 5 mm quartz NMR tubes were used, containing 500 µL of the pH-adjusted sample solution as well as a coaxial capillary insert filled with D_2O (Deutero, 99.95 % D, containing 0.03 % sodium 3-trimethylsilyl-propionate, TMSP-d_4) for deuterium lock along with one droplet of 85 % H_3PO_4 (Roth, p.a.) for external referencing. ^{1}H NMR spectra were acquired by accumulating 32 transients, applying a 2 s pre-saturation pulse on the water resonance for water signal suppression, and referenced relative to the methyl signal of TMSP-d_4 in D_2O with $\delta_H = 0.00$ ppm. Broadband ^{1}H-decoupled ^{31}P NMR spectra were obtained after excitation by a 30° pulse (4.16 µs) and acquisition of the FID for 2 s. The relaxation delay (d1) between two excitations was 3 s. All measurements were performed under ambient atmosphere at (25 ± 1)°C.

Attenuated Total Reflection Fourier-transform Infrared spectroscopy (ATR FT-IR)

ATR FT-IR spectroscopy was performed using a Vertex 80/v vacuum spectrometer from Bruker Optics Inc. equipped with a mercury cadmium telluride detector. The ATR accessory unit (DURA SamplIR from Smiths Inc.) consisted of a horizontal diamond with nine internal reflections and an

incidence angle of 45°. For the measurements of the liquid samples, a flow cell with a total volume of 200 µL was used to reduce external thermal interferences. IR spectra were recorded in the range of 1800 – 700 cm^{-1} at a spectral resolution of 4 cm^{-1}. All IR experiments were performed under ambient atmosphere at (25 ± 1) °C.

The IR experiments were performed at distinct pH values in the range from 1 to 12. At each pH, single beam spectra of a series of solutions containing the background electrolyte, the ligand, the Eu complexes of the ligand, and finally again the background electrolyte were subsequently recorded by exchanging the liquids in the flow cell via syringes. Absorption spectra of the ligand and the corresponding Eu(III) complexes were calculated from spectra of the respective solutions and the background electrolyte according to Beer's law. In analogy, difference spectra of samples containing the Eu(III) complexes and the ligand were calculated. These spectra explicitly reveal the spectral differences of the ligand occurring upon complexation with Eu(III) and potentially show positive and negative bands representing vibrational modes of the complexes and the pure ligand, respectively. All spectra shown represent the average of at least four spectra calculated from single beam spectra averaged out of 256 scans.

The solutions of the ligand and the Eu(III) complexes contained 1 mM HEDP and M:L of 1:1 and 1:3, respectively. The ionic strength in all samples was set to 0.1 M (NaCl). Spectra of Eu(III) containing HEDP solutions in the pH range from 2 to 12 showed contributions from precipitations which are currently under investigation. Therefore, only IR spectra of the homogeneously liquid phases are presented.

Time-resolved laser-induced fluorescence spectroscopy (TRLFS)

TRLFS measurements were performed using the Ekspla NT230 Laser, providing ~1 mJ per pulse 5 ns pulses at a 50 Hz repetition rate. The excitation wavelength was set to 394 nm with a constant time window of 1 ms. The sample temperature was set to (25 ± 1) °C and controlled by a cuvette holder (Quantum Northwest). Emitted light was collected perpendicular to the excitation beam and brought to the spectrograph via a light guide. The spectrograph (Andor, SR-303i-A, input slit width 300 µm) is equipped with an ICCD (Andor iStar, DH320T-18U-63, gate width 1 ms, linear increasing steps 3+3*x, kinetic series length 30, gain 4095) and the luminescence spectra were recorded in the wavelength range from 500 to 760 nm (grating: 300 lines/mm with a resolution of 0.25 nm). To record time-dependent luminescence spectra, the delay time between laser pulse and camera control was sampled in dynamic time intervals (usually between 1 and 301 µs).

Initial measurements revealed differences between spectra of samples measured directly after preparation and those of samples with equilibration time of 1 – 5 days. This issue is ascribed to the low solubility within the Eu(III)–HEDP system described in section 2.2. Therefore, all samples were measured after an equilibration time of at least 24 h.

Mass spectrometry with electrospray ionization (ESI-MS)

ESI-MS was conducted using an ESI-Source (Thermo Scientific Nanospray Flex Ion Source) coupled to an Orbitrap mass spectrometer (Thermo Scientific Orbitrap Elite). 10 µL of each sample solution were transferred into the nanospray emitters (New Objective PicoTip Emitter). Mass spectra were acquired in both positive and negative ion mode in the range of m/z 100-1000 and at a capillary temperature of 275 °C. All measurements were taken with a mass resolution (FWHM) of 120,000 at *m/z* 400.

Data processing and analysis

NMR spectra were processed and evaluated with MestReNova (version 6.0.2., Mestrelab Research S.L. [54]). Creation of graphs for numerical data visualization and data fitting by non-linear sigmoidal dose-response fit algorithm was performed with Origin 2019 (version 9.6.0.172, OriginLab Corporation). The procedure for the determination of pK_a values is adapted from [55]. The pK_a constants were obtained from the inflection points of sigmoidal dose-response fits to the pH-dependent ^{1}H and ^{31}P

chemical shift data. Extrapolation of the protonation constants for zero ionic strength, pK_a^0, was calculated with ionic strength corrections using Specific Interaction Theory (SIT) [56].

Speciation calculations of HEDP in water were carried out with HySS2006 (version 4.0.21, Protonic Software [57]) using the ligand concentration, the pH, and the determined pK_a values as input parameters.

All absorption (IR) and emission (TRLFS) spectra were processed and analyzed with Origin 2020b (version 9.7.5.184, OriginLab Corporation) to obtain peak positions. If necessary, TRLFS spectra were baseline corrected and the steady–state spectra normalized to the peak area of the $^5D_0 \rightarrow {}^7F_1$ band because this transition is considered to be independent from the chemical environment of the metal ion [58-60]. The intensity ratio between the electric dipole transition $^5D_0 \rightarrow {}^7F_2$ and the magnetic dipole transition $^5D_0 \rightarrow {}^7F_1$ ($R_{E/M}$) and the luminescence lifetime of emitting species was also determined with Origin. Details on this and the respective equations are given elsewhere [32]. From the luminescence lifetime, the number of water molecules in the first coordination shell of the metal ion was calculated using the equation given by [48] with τ inserted in ms:

$$n_{H2O} \pm 0.5 = 1.07/\tau - 0.62. \quad (1)$$

Formation constants and emission spectra of single Eu(III)–HEDP species were determined independently using Specfit (Spectrum Software Associates) [61] and parallel factor analysis (PARAFAC) using the N-way toolbox for Matlab [62] (for more details refer to [50, 63]. Both factor analysis programs decompose spectra of mixtures into their components based on the spectroscopic properties of each species. These properties vary in dependence on the metal and ligand concentrations as well as the pH. Furthermore, both programs calculate reasonable complex stability constants. The successful application of these software was demonstrated in earlier studies on the complexation of Eu(III) with various ligands [32,33, 50, 63, 64].

Based on the identified species, the formation constants for the overall (log β_{pqr}) and direct reactions (log K_{pqr}) were calculated, where p, q, and r denote the number of metal ions, ligand molecules, and protons, respectively. The log K_{pqr} values were calculated according to the mass action law for the following equations:

$$Eu^{3+} + H_2L^{2-} \rightleftharpoons EuH_2L^+ \text{ for log } K_{112}, \quad (2)$$

$$Eu^{3+} + 2\ H_2L^{2-} \rightleftharpoons EuH_4L_2^- \text{ for log } K_{124}, \quad (3)$$

$$Eu^{3+} + HL^{3-} \rightleftharpoons EuHL^0 \text{ for log } K_{111}, \text{ and} \quad (4)$$

$$Eu^{3+} + L^{4-} \rightleftharpoons EuL^- \text{ for log } K_{110} = \text{log } \beta_{110}. \quad (5)$$

Consequently, the log β_{pqr} values were calculated according to the mass action law for the following equations:

$$Eu^{3+} + L^{4-} + H^+ \rightleftharpoons EuHL^0 \text{ for log } \beta_{111}, \quad (6)$$

$$Eu^{3+} + L^{4-} + 2\ H^+ \rightleftharpoons EuH_2L^+ \text{ for log } \beta_{112}, \text{ and} \quad (7)$$

$$Eu^{3+} + 2\ L^{4-} + 4\ H^+ \rightleftharpoons EuH_4L_2^- \text{ for log } \beta_{124}. \quad (8)$$

Extrapolation of the complex formation constants for zero ionic strength, log K^0 and log β^0, was calculated with ionic strength corrections using Specific Interaction Theory (SIT) [57].

Data analysis of ESI-MS spectra was performed using Thermo Scientific FreeStyle and an in-house software solution named MARI [65]. Species of interest were identified based on their theoretical mass and specific isotopic pattern. Mass accuracy was 5 ppm or better for all species identified. Typical adducts and artifacts from the ESI-process were considered in the analysis.

Density functional theory (DFT)

DFT calculations were performed using Gaussian 16 program (Gaussian Inc.) [66]. Geometries were optimized in the aqueous phase (eps = 78.3553) at the B3LYP level using the PCM solvation model with UFF (universal force field) radii. The large core effective core potential (LC–ECP) as well as the corresponding basis sets suggested by [67] was used on Eu(III). For P, C, O, and H, all-electron valence triple–ζ basis set plus polarization and diffuse functions (6-311G*) have been used [68]. The LC–ECP used in this study is specifically designed for Eu(III) incorporating six unpaired 4f electrons into the core potential, thereby enabling closed-shell calculations on Eu^{3+} which has the formal electronic configuration of the septet state. This simplification is justified by the 4f orbital being strongly contracted, shielded by valence orbitals, and not actively participating in chemical bonding between Eu(III) and a ligand. The same methodology was successfully used in previous publications [32, 69].

The standard Stuttgart/Dresden-type (SDD) pseudopotentials and basis sets were very difficult to get convergence. The final geometries were confirmed to be the energy minimum through vibrational frequency analysis where no imaginary frequency was found to be present. The vibrational spectra were fitted with the halfwidth of 8 cm^{-1} at half-height using the calculated harmonic frequencies and IR intensities.

As a first step, the energetically most stable HEDP ligand species in deprotonation state were studied and the respective IR spectrum calculated. Then, the complexation with Eu(III) was investigated and the energetically most stable complex structures with different stoichiometry and HEDP ligand species were determined.

Characterization of the HEDP ligand (detailed)

NMR spectroscopy

The pH-dependent ^{1}H and ^{31}P NMR spectra are shown in Figure S1. The HEDP methyl group's ^{1}H signal appears as a triplet owing to the coupling to the two ^{31}P spin-½ nuclei. When the pH is increased, it shows a continuous shift to smaller chemical shift values owing to the successive shielding of the methyl ^{1}H nuclei upon increasing HEDP's anionic charge due to stepwise deprotonation. The ^{31}P signal features only one peak arising from the phosphonate groups. Because of HEDP's symmetry and the rapid proton exchange at the phosphonate groups, the two individual P atoms are equivalent and cannot be discriminated via NMR in aqueous solution [70, 71]. Since the ^{31}P NMR spectra are ^{1}H-decoupled, the actual quartet, arising from the coupling to the three methyl ^{1}H spin-½ nuclei, collapses to a singlet. In contrast to the ^{1}H spectrum, the ^{31}P spectra show a more complex pH-dependency as already observed for other phosphonic acids [55, 72, 73]. Both ^{1}H and ^{31}P spectra correlate nicely throughout the whole pH-titration series (see Figure S2) and exhibit plateaus corresponding to nearly exclusively existing species, e.g. H_2L^{2-} for $3.5 < pH < 6$ and HL^{3-} for $8 < pH < 10$. Up to pH 12.75, there is no indication of hydroxyl group deprotonation.

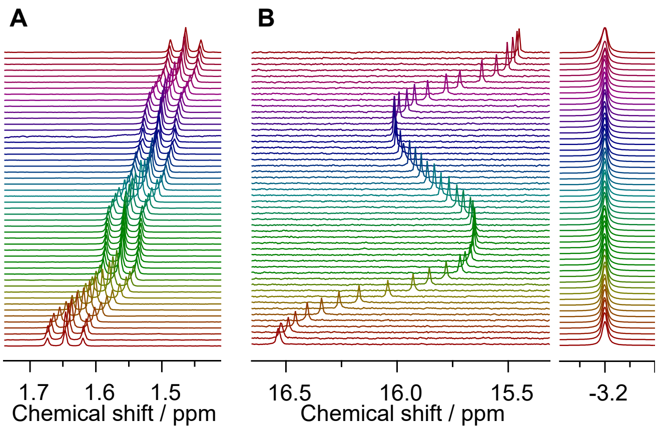

Figure S1: ^{1}H (A) and ^{31}P NMR (B) pH-titration series of 1 mM HEDP at $I = 0.1$ M (NaCl, KCl) and room temperature in the pH range from 0.5 to 13 (from bottom to top). Depicted on the right is the ^{31}P signal of the external chemical shift reference.

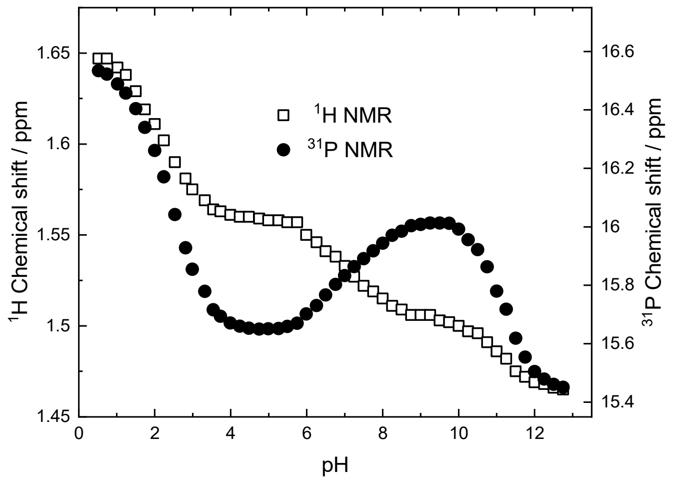

Figure S2: ^{1}H (methyl group) and ^{31}P NMR chemical shift values of HEDP in dependence on pH, based on the spectra obtained from 1 mM HEDP aqueous solutions at I = 0.1 M and (25 ± 1) °C in the range of 0.5 < pH < 13.

Using our pK_a values at I = 0.1 M (NaCl) which are in good agreement with the literature data (see Table S1), the aqueous speciation was calculated and is provided as Figure S3. HEDP is deprotonated stepwise and there are pH ranges of exclusive existence for almost all protonated ligand species. Hence, the fully protonated acid (H_4L) dominates at pH < 1, the singly deprotonated H_3L^- species around pH 2, the doubly deprotonated H_2L^{2-} anion at 4 < pH < 6, the trifold deprotonated HL^{3-} ligand at 8 < pH < 10, and the fully deprotonated L^{4-} anion at pH > 12. These pH ranges of exclusive existence have been taken into account for the subsequent ATR-FT-IR measurements of HEDP as well as for the metal ion complexation studies.

Table S1: Overview and comparison of pK_a values for HEDP in literature at 25 °C.

pK_a					method	I	reference
**1**	**2**	**3**	**4**	**5**			
2.00	2.90	7.23	11.37	11.96	potentiometric	~ 0.04 M (KOH)	[38]
**1.36 ± 0.31**	**2.46 ± 0.11**	**6.93 ± 0.09**	**11.12 ± 0.30**	**> 14**	**^{1}H + ^{31}P NMR**	**0.1 M (NaCl/KCl)[1]**	**present work**
-	2.80 ± 0.02	7.00 ± 0.02	11.16 ± 0.07	-	potentiometric	0.1 M $(CH_3)_4NCl$	[36]
-	2.31	6.99	10.93	-	potentiometric	0.1 M $(C_2H_5)_4NClO_4$	[14]
1.49 ± 0.20	2.50 ± 0.02	6.80 ± 0.01	10.52 ± 0.01	-	potentiometric	0.1 M $NaClO_4$	[74]
1.21 ± 0.12	2.55 ± 0.03	6.87 ± 0.02	10.66 ± 0.01	-	^{31}P NMR	0.1 M NaCl	[73]
< 2	2.5 ± 0.2	6.89 ± 0.01	10.60 ± 0.02	-	potentiometric	0.1 M KCl	[13]
1.70	2.47	7.28	10.29	11.13	potentiometric	0.1 M KCl	[39]
-	2.59	6.87	10.98	-	potentiometric	0.1 M KNO_3	[15]
-	2.77 ± 0.01	6.99 ± 0.01	11.23 ± 0.01	-	potentiometric	0.1 M KNO_3	[16]
< 1	2.54 ± 0.05[2]	6.97 ± 0.05	11.41 ± 0.05	-	potentiometric	0.5 M $(CH_3)_4NCl$	[35]
1.21 ± 0.02	2.34 ± 0.01	6.44 ± 0.00	10.08 ± 0.00	-	potentiometric	1 M KCl	[37]
1.56 ± 0.01	2.20 ± 0.01	6.20 ± 0.01	9.12 ± 0.01	-	potentiometric	2 M $NaNO_3$	[23]
				> 14.6		3.4 M $(CH_3)_4NCl$	[40]

[1] For pH < 7, NaCl was used, for pH > 7 KCl was used in order to avoid alkali error at high pH values using a glass electrode (3 M KCl).

[2] The pK_{a2} was determined with the assumption of pK_{a1} = 1.

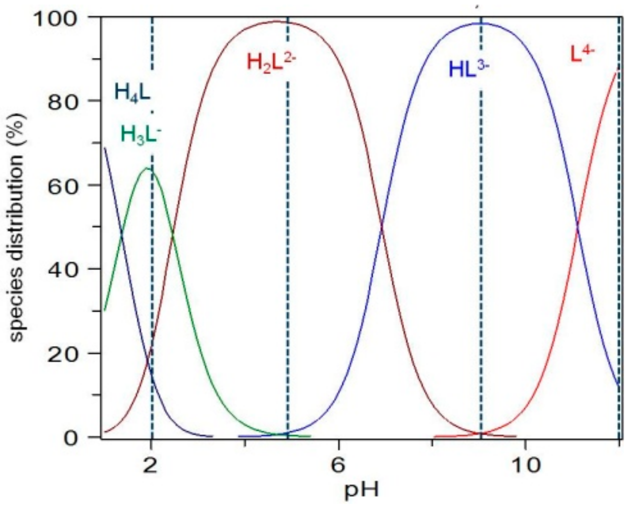

Figure S3: Species distribution of pure HEDP in aqueous solution at *I* = 0.1 M (NaCl) from pH 1 to 12 using the p*K*a values determined in this study.

IR spectroscopy and theoretical DFT calculations

The IR spectra of HEDP measured within the range of 0.5 < pH < 13 are given in Figure S4 those of the distinct HEDP species in Figure S5A. In addition to the experimental data, the IR spectrum of each HEDP species was also calculated using DFT (Figure S5B).

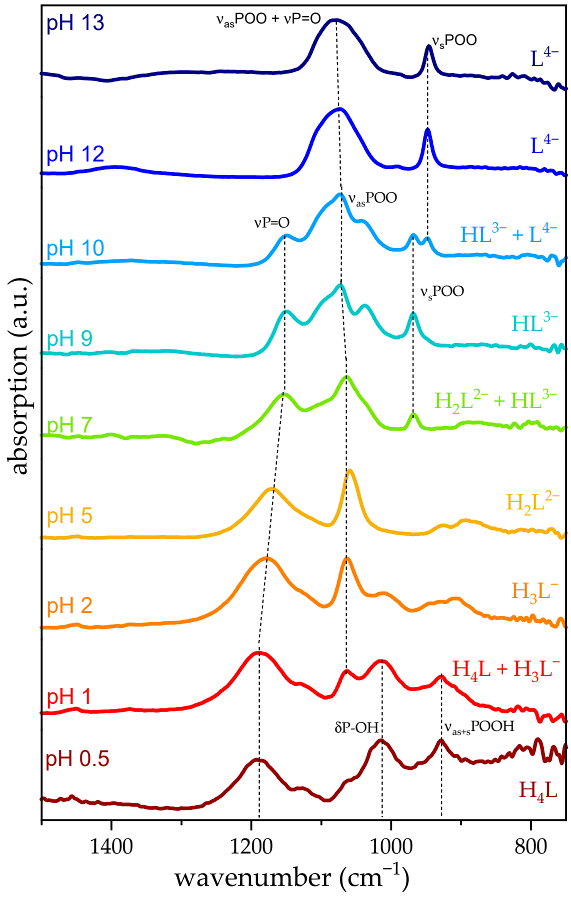

Figure S4: IR absorption spectra of HEDP in dependence on the pH over the pH range from 1–13 along with the respectively dominating ligand species.

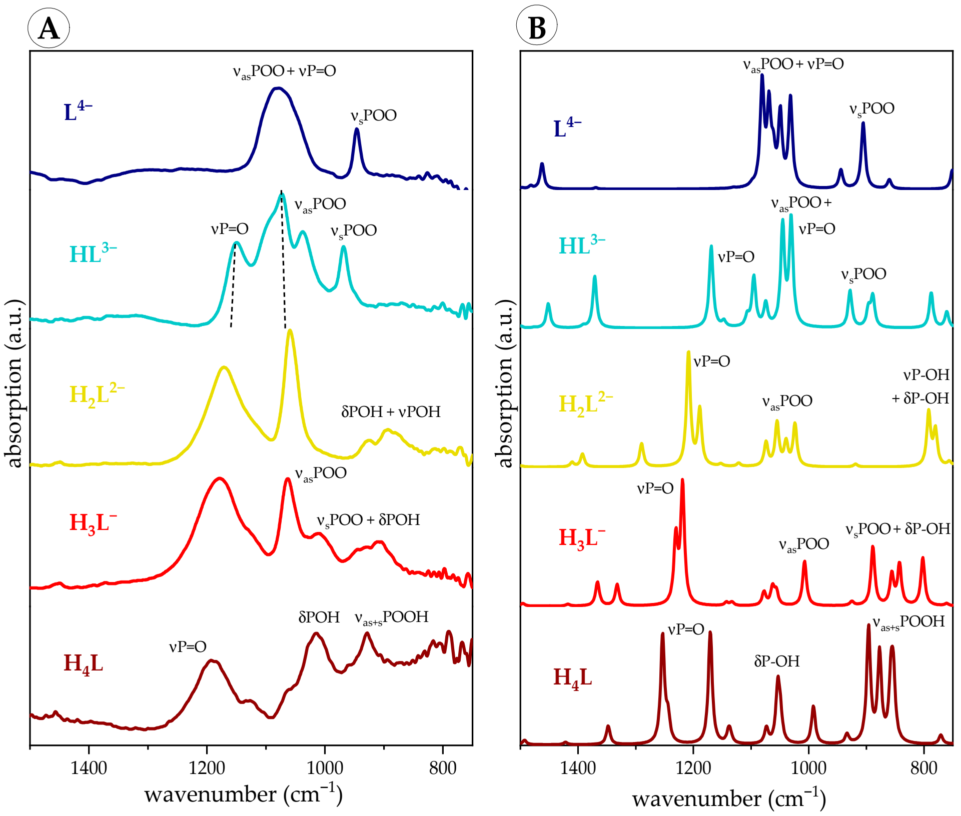

Figure S5: IR absorption spectra of the several HEDP species at different pH: A) experimental data measured in 5 mM HEDP at *I* = 0.1 M (NaCl) as well as (25 ± 1) °C and B) corresponding spectra obtained from DFT calculations.

A tentative assignment of the vibrational bands is given in Table S2. All IR spectra shown are dominated by strong bands which can be assigned to vibrational modes of the phosphonate groups. However, signals associated with the hydroxyl group might occur in the same spectral region. The stepwise deprotonation of HEDP with increasing pH is reflected by strong alterations of the vibrational bands assigned to the phosphonate groups. In general, with increasing pH, the ν_{as}(POO) and ν_s(POO) modes and the ν(P=O) mode are shifted to higher and lower wavenumbers, respectively, until the ν(P=O) and the ν_{as}(POO) modes are observed as one broad band at highly alkaline pH. In agreement with NMR measurements, a deprotonation of the hydroxyl group was not observed up to pH 13. In such a case a characteristic strong band representing the CO stretching mode at around 1000 cm^{-1} would be expected as reported for citric acid [32].

Table S2: Tentative assignment of the IR absorption bands of HEDP.

wavenumber (cm^{-1})[1]					
H_4L (pH < 1)	H_3L^- (pH 2)	H_2L^{2-} (pH 4 – 6)	HL^{3-} (pH 8 – 10)	L^{4-} (pH > 11)	**assignment**[2]
1655 (w)	1630 (w)	1655 (w)	1657 (w)	1636 (s)	δ(H_2O)
–	1450 (w)	1449 (w)	–	–	δ(CH_3)
1190 (s)	1179 (s)	1171 (s)	1151 (m)	–	ν(P=O)
–	1063 (m)	1059 (m)	1072 (s)	1078 (s)	ν_{as}(POO)
1014 (m)	1012 (w)	–	–	–	δ(POH)
928 (w)	908 (w)	–	968 (m)	946 (m)	ν_s(POO)
–	155	–	104	132	$\Delta\nu_3$

[1] s – strong, m – medium, w – weak.

[2] δ – deformation vibration, ν – stretching vibration, ν_s/ν_{as} – symmetric/antisymmetric stretching vibration, $\Delta\nu_3 = \nu_{as} - \nu_s$ (spectral splitting of ν_{as} and ν_s).

Solubility of europium(III) in the presence of HEDP (detailed)

The as the ICP-MS data and solubility curves of pH-titration series are provided in Tables S3 and S4 as well as Figure S6.

Table S3: Solubility in the Eu(III)–HEDP system at **10^{-6} M** Eu(III) at I = 0 and (25 ± 1) °C.

M:L	*pH*	*Eu_{sol} (%)* *I = 0 M*
1 : 1	1 – 4	80 – 100
	4 – 9	5 – 20
	9 – 12	50 – 120
1 : 3	1 – 4	80 – 100
	4 – 8	5 – 45
	8 – 12	45 – 80
pH	**M:L**	**Eu_{sol} (%)** ***I* = 0 M**
2	1 : 0.1 – 1 : 10	90 – 100
5	≤ 1 : 0.2	65 – 70
	1 : 0.6 – 1 : 3	10 – 25
	1 : 5 – 1 : 10	35 – 40
11.5	1 : 0.1 – 1 : 0.6	35 – 70
	1 : 0.8 – 1 : 10	80 – 95

Table S4: Solubility in the Eu(III)–HEDP system at **10^{-3} M** Eu(III) at I = 0.1 M (NaCl) and (25 ± 1) °C.

M:L	*pH*	*Eu_{sol} (%)* ***I = 0.1 M (NaCl)***
1 : 1	1	100
	2.5 – 9	0 – 4
	12	94
1 : 3	1	52
	2.5 – 9	0 – 5
	12	100

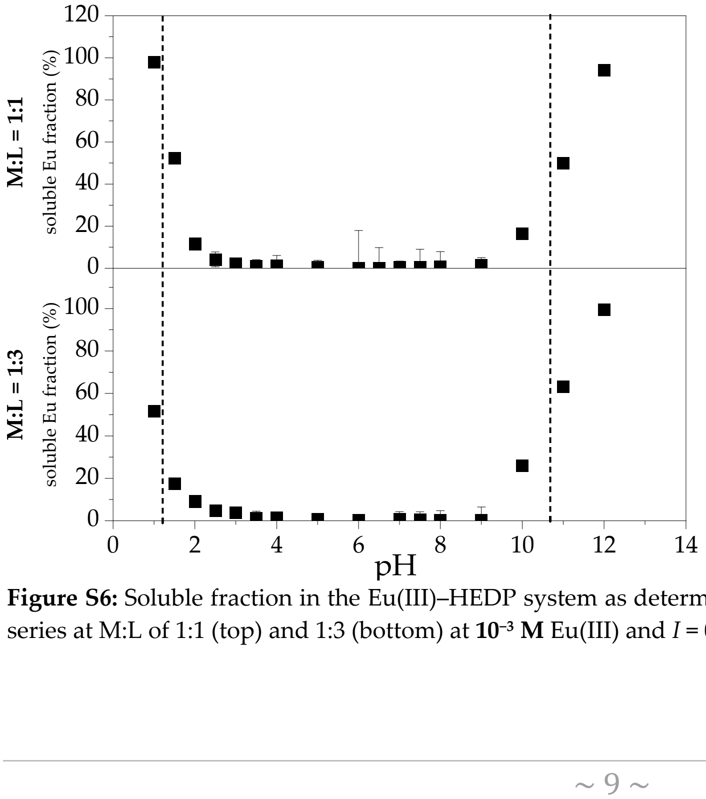

Figure S6: Soluble fraction in the Eu(III)–HEDP system as determined by ICP-MS in supernatants of pH-titration series at M:L of 1:1 (top) and 1:3 (bottom) at **10^{-3} M** Eu(III) and I = 0.1 M (NaCl) and at (25 ± 1) °C.

Structural investigations on soluble europium(III)–HEDP complexes

The results from ESI-MS are provided in Figure S7 and Table S5.

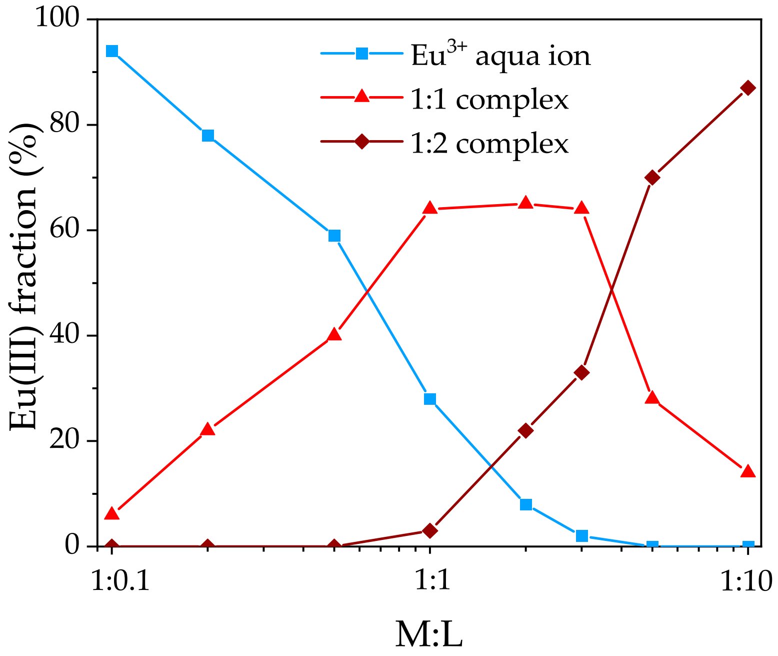

Figure S7: Stoichiometry and distribution of complexes formed in the Eu(III)–HEDP system at pH 2 in dependence on the given M:L (10^{-4} M Eu(III), without background electrolyte and at (25 ± 1) °C) as measured with ESI-MS using positive mode.

Table S5: Mass-to-charge peaks of several Eu(III) species in the Eu(III)–HEDP system measured by ESI-MS at pH 2 as well as the corresponding detection mode.

Eu(III) species	*detected ESI-MS species*	m/z_0[1]	*detection mode*
Eu^{3+} aqua ion	$Eu^{II}OH^+$	169.9	positive
	$Eu^{III}(OH)_2^+$	186.9	positive
	$Eu(OH)_2(H_2O)^+$	204.8	positive
EuH_3L^{2+}	$Eu(H_3L)(H_2O)Cl^+$	410.9	positive
	$Eu(H_3L)(H_2O)_2Cl^+$	429.9	positive
EuH_2L^+	EuH_2L^+	356.9	positive
	$Eu(H_2L)(H_2O)^+$	374.9	positive
	$Eu(H_2L)(H_2O)_2^+$	392.9	positive
$Eu(H_3L)_2^+$	$Eu(H_3L)_2^+$	592.9	positive
	$Eu(H_3L)_2(H_2O)^+$	430.8	positive
$Eu(H_2L)_2^-$	$Eu^{II}(H_2L)_2^-$	280.4	negative
	$Eu^{III}(H_2L)_2^{2-}$	560.8	negative

[1] Mass to charge ratio of the most prominent peaks including adducts with H_2O, OH^-, H^+ and Cl^- but excluding those with Na^+ and K^+.

IR spectroscopic data at pH 0.5 and 12.5 are provided in Figure S8.

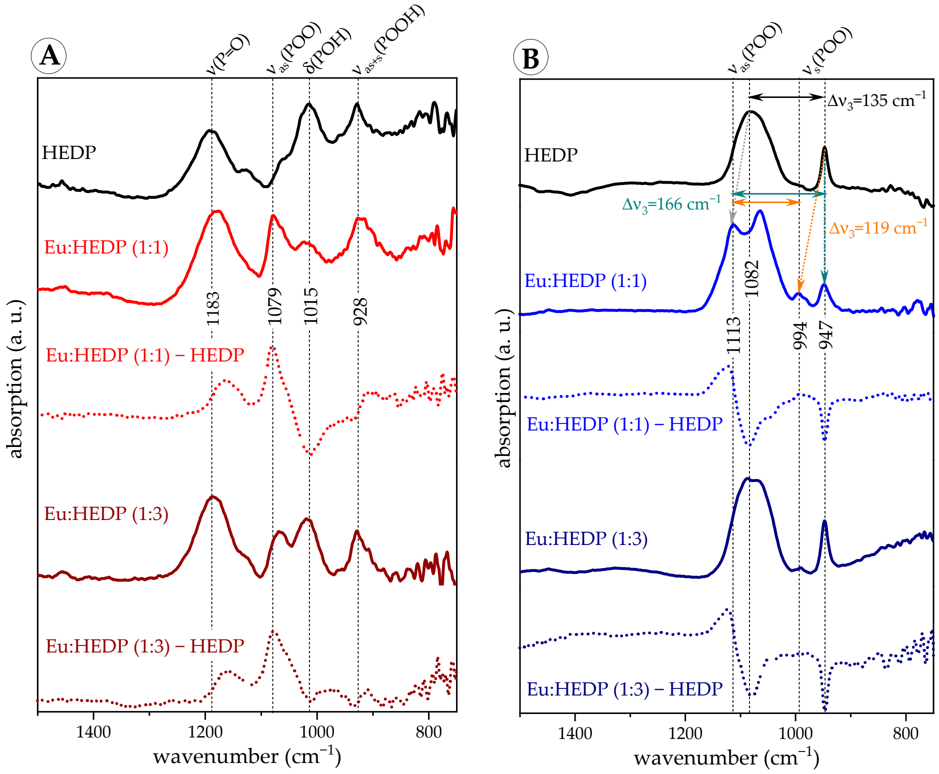

Figure S8: IR spectra of aqueous solutions of 5 mM HEDP and of Eu-HEDP at two different M:L ratios, i.e., 1:1 and 1:3, recorded at pH 0.5 (A) and pH 12.5 (B), at I = 0 M and (25 ± 1) °C. For clarity, difference spectra were calculated out of single beam spectra recorded of HEDP in the presence and absence of Eu (third and fifth traces) explicitly showing the spectral differences occurring upon complexation. Spectra of aqueous HEDP are shown as reference on top. (Spectra at I = 0.1 M (NaCl) are nearly identical.)

At pH 12.5 the spectral splitting of the ν_3 mode upon Eu(III) complexation can be interpreted in two ways: (*i*) ν_{as}(POO) is blueshifted to 1113 cm^{-1} and ν_s(POO) remains at 947 cm^{-1} resulting in a slightly increased $\Delta\nu_3$ of about 166 cm^{-1} or (*ii*) both split modes are blueshifted to 1113 and 994 cm^{-1} resulting in a slightly decreased $\Delta\nu_3$ of about 119 cm^{-1}. In both cases, the ν_s mode is not shifted in comparison to the reference spectrum where Na^+ is the counterion. A bidentate coordination is however expected to generate a redshift of the ν_{as}(POO) mode as recently reported [39]. Therefore, we conclude that the shifts observed reflect the coordination of HEDP to Eu(III) in the same binding mode as to Na^+ which is most likely a monodentate coordination.

NMR spectroscopic data at pH 0.5 and pH 12.5 are provided in Figures S9 and S10.

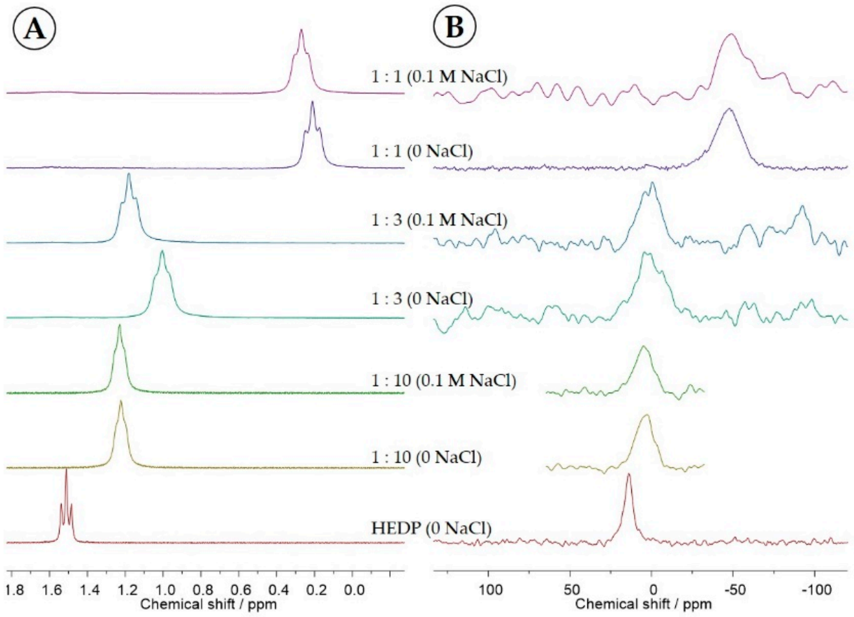

Figure S9: ^{1}H (A) and ^{31}P{^{1}H} NMR spectra (B) of 5 mM HEDP in aqueous solution containing 10% D_2O at pH 0.5, acquired at 25 °C, in absence (0 NaCl) or presence of 0.1 M NaCl, and varying M:L as stated with the spectra. Depicted on the bottom are the respective reference spectra of the pure HEDP ligand at pH 0.5. Note that the 1:1 (0 NaCl) spectrum was acquired with 12000 scans as compared to only 1024 scans as for the other ^{31}P spectra, hence showing a better signal to noise ratio. The water signal was suppressed by a pre-saturation sequence.

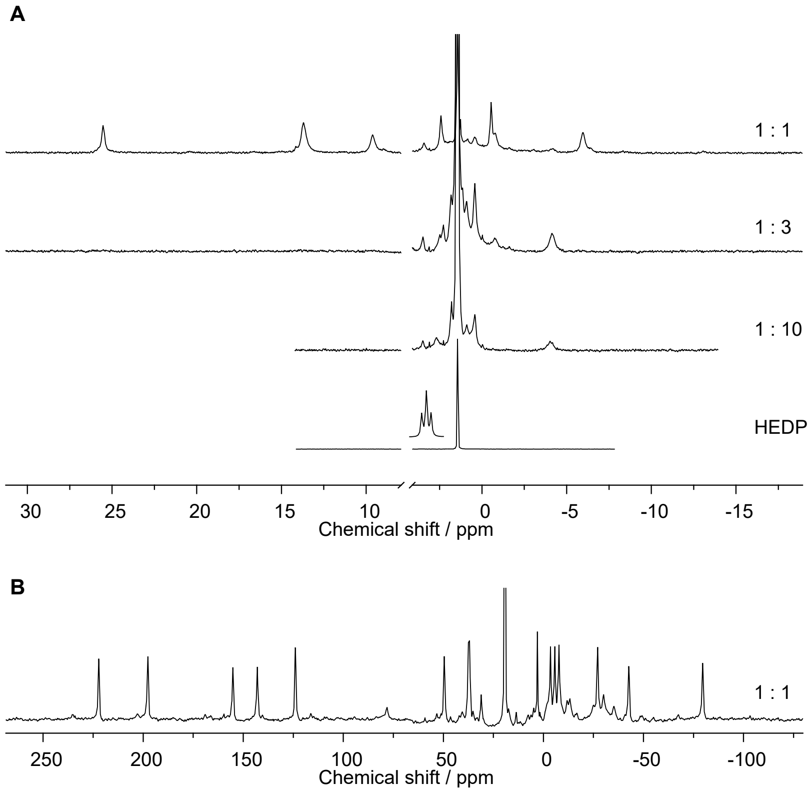

Figure S10: (A) ^{1}H NMR spectra of 5 mM HEDP in aqueous solution containing 10% D_2O at pH 12.5, acquired at (25 ± 1) °C, without background electrolyte, and varying M:L as stated with the spectra. The water signal was suppressed by a pre-saturation sequence. Depicted on the bottom is the respective reference spectrum of the pure HEDP ligand at pH 12.5 along with a magnification showing the triplet fine structure as insert. Only spectral regions of interest are shown. (B) ^{31}P{^{1}H} NMR spectrum of an aqueous solution containing 10% D_2O at pH 12.5, 5 mM each in Eu(III) and HEDP, corresponding to the top spectrum in (A).

Besides the fact that the observed signals are molar fraction-weighted averages of free and Eu(III)-bound HEDP, europium's 4f electrons' unpaired spins cause paramagnetic-induced effects, i.e. shifts as well as line broadening due to enhanced relaxation.

In comparison to the HEDP reference spectrum at pH 12.5, in presence of Eu(III) several new signals appear, both downfield and upfield from unbound HEDP, indicating (*i*) some interaction between Eu(III) and HEDP (at pH 12.5 most likely as L^{4-}), and (*ii*) soluble complex species.

Especially for equimolar M:L both ^{1}H and ^{31}P NMR spectra reveal unique signals, all of comparable intensity. The latter fact speaks against a multitude of species coincidently existing in similar quantity. Instead, it rather seems that either one complex occurs as different isomers or the species is polynuclear and thus of higher stoichiometry involving several spectroscopically unique ligand molecules. Taking into account the high concentrations applied for IR and NMR spectroscopies, the formation of polynuclear species is very likely. The fourteen distinct ^{31}P signals imply that this complex species comprises seven HEDP ligand molecules that can be discriminated (only) by NMR spectroscopy, owing to the symmetry as well as pseudocontact and/or Fermi contact contributions from the involved Eu(III) ions the number of which is yet unclear, however, likely amounts to three to seven. In either case, with the seven ligand molecules the polynuclear species is highly negatively charged and hence of high solubility.

Thermodynamic studies of the europium(III)–HEDP system (detailed)

TRLFS data at 10^{-6} and 10^{-5} M Eu(III) from HEDP concentration series

TRLF spectra of HEDP concentration series at constant pH values are provided in Figures S11 and S12.

In the acidic region at pH 2, up to a M:L of about 1:0.6, all parameters correspond to the Eu^{3+} aqua ion. At nearly equimolar M:L up to tenfold ligand excess, the raw luminescence intensity constantly increases, the $R_{E/M}$ is enhanced to 1.2, and the luminescence lifetime is prolonged to about 140 µs. This Eu(III)–HEDP species is referred to as *complex 1* and was verified at 10^{-5} M Eu(III), at which complexation with HEDP even starts at tenfold ligand deficit with formation of the same complex species. Additionally, luminescence spectra at 10^{-5} M Eu(III) and ligand excess indicate the formation of a further Eu(III)–HEDP species, *complex 2*, exhibiting a higher $R_{E/M}$ and a longer luminescence lifetime than *complex 1*.

In the near-neutral region at pH 5.5, *complex 3* is formed and spectral alterations are observed even at the lowest ligand concentration, i.e. tenfold HEDP deficit. At M:L = 1:>0.6, the normalized TRLF spectra are identical and exhibit a significantly enhanced $R_{E/M}$ of 4.5 as well as a luminescence lifetime of (177 ± 15) µs. Interestingly, the raw luminescence intensity of the samples increases with increasing ligand concentration till a M:L of 1:2 but drops at higher ligand excess. Since the spectral parameters remain unaltered this reflects the solubility issue described earlier and the precipitation of *complex 3*. At 10^{-5} M Eu(III), further significant spectral alterations are observed at twofold and higher HEDP excess indicating the formation of a *complex 4* being hardly soluble, too. The TRLF spectrum of this species is characterized by a $R_{E/M}$ of 4.9, a pronounced splitting of both the $^{7}F_{1}$ and $^{7}F_{2}$ bands and a luminescence lifetime of (371 ± 15) µs.

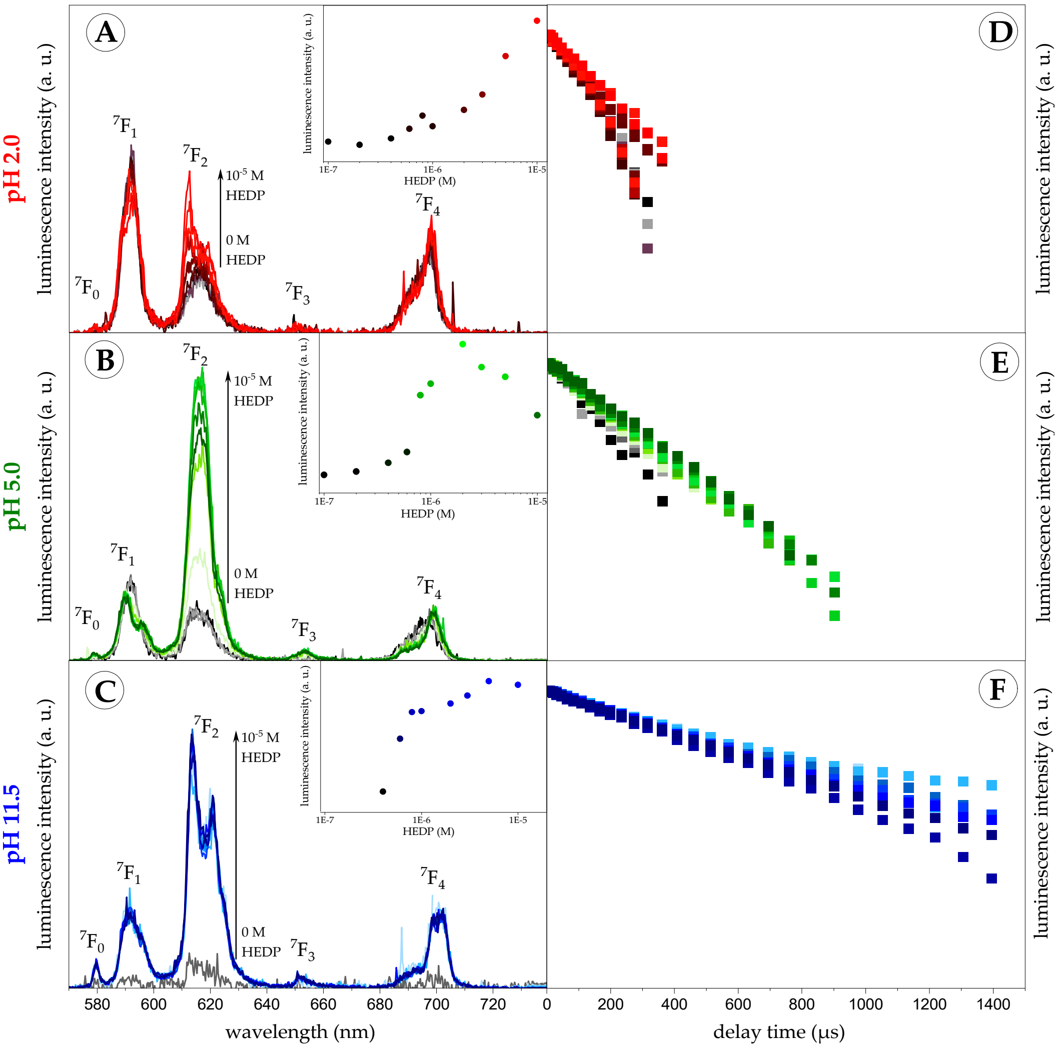

Figure S11: Steady-state luminescence spectra (A–C) and luminescence decay curves (D–F) of **10^{-6} M** Eu(III) at constant pH 2.0 (A and D), 5.0 (B and E), and 11.5 (C and F), without background electrolyte and at (25 ± 1) °C in dependence on HEDP concentration (steady-state spectra are normalized to the $^7F_1$ band area and decay curves are normalized to the luminescence intensity at *t* = 0). Inserts show the HEDP concentration-dependent raw luminescence intensity.

In the alkaline region at pH 11.5, due to Eu(III) hydrolysis and the formation of non-luminescent $Eu(OH)_3$ no TRLF spectrum is detected for M:L < 1:0.2. Starting at M:L of 1 : 0.4, a luminescence spectrum is recorded which is distinct from all Eu(III)–HEDP species previously described. Raw luminescence intensity is increasing with increasing HEDP concentration and reaches a plateau around fivefold ligand excess indicating no solubility issues. Normalized TRLF spectra are identical and indicate the formation of a further unique *complex 5* with a $R_{E/M}$ of 3.5 and a luminescence lifetime of (385 ± 14) µs. At 10^{-5} M Eu(III), this species is formed only at ligand deficit up to equimolar M:L. At HEDP excess, the luminescence spectra are identical to those of *complex 4* at pH 5.5 and a decrease in the measured luminescence intensity indicates precipitation of this species.

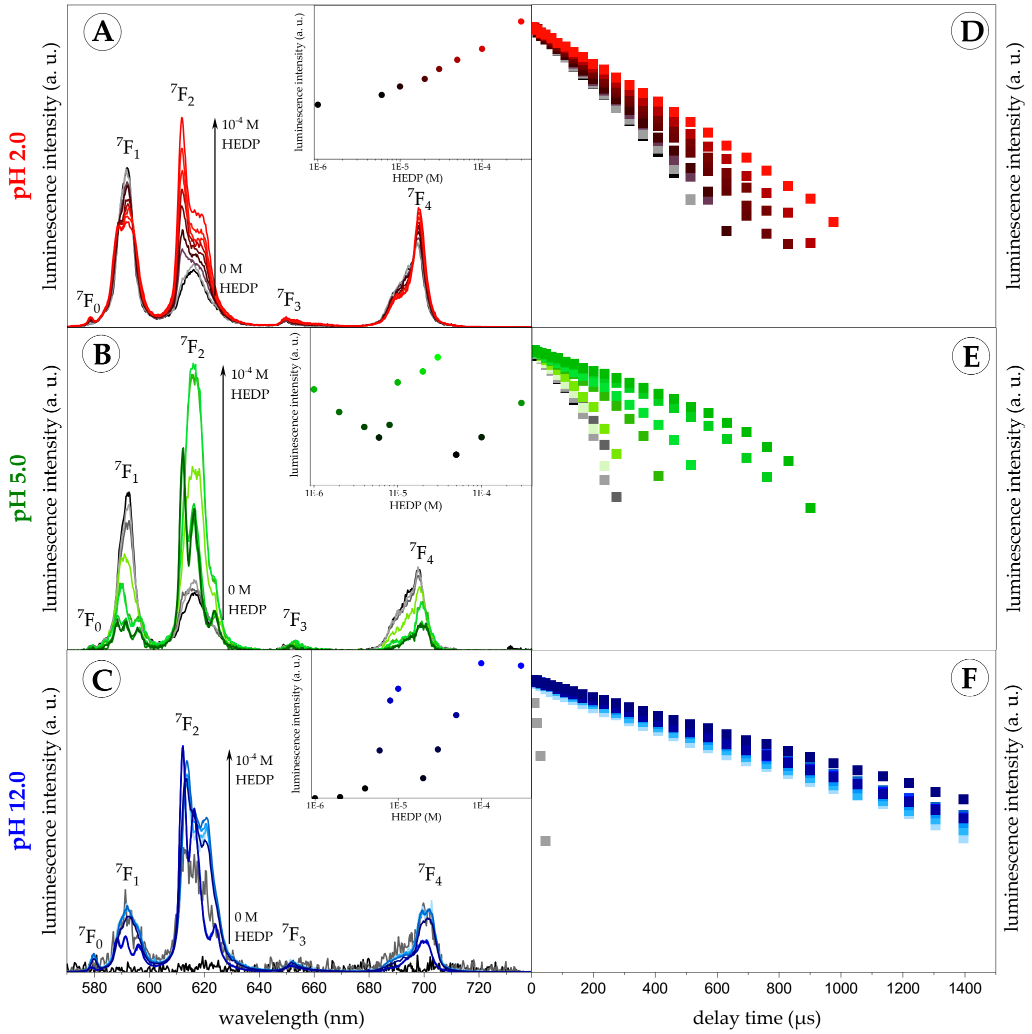

Figure S12: Steady-state luminescence spectra (A–C) and luminescence decay curves (D–F) of the soluble fraction (supernatants) of **10^{-5} M** Eu(III) at constant pH 2.0 (A and D), 5.0 (B and E), and 12.0 (C and F) as well as I = 0.1 M (NaCl) and at (25 ± 1) °C in dependence on HEDP concentration (steady-state spectra are normalized to the $^7F_1$ band are, and decay curves are normalized to the luminescence intensity at t = 0.). Inserts show the HEDP concentration-dependent raw luminescence intensity.

TRLFS data at 10^{-6} and 10^{-5} M Eu(III) from pH series

TRLF spectra of pH series at constant M:L of both 1:1 and 1:3 are provided in Figures S13 and S14. In general, at 10^{-6} M Eu(III), series for both M:L give the same results. Under very acidic conditions the Eu^{3+} aqua ion is the exclusive species. Eu(III) complexation with HEDP starts as of pH 2. Along the regimes of acidic, near-neutral, and alkaline pH, the *complexes 1*, *3*, and *5* are consecutively formed. Eu(III) hydrolysis competes with HEDP complexation in the range of pH 7.5 – 10.0 and above pH 12.0. Remarkably, the raw luminescence intensity is noticeably reduced in the near-neutral pH range compared to that at both lower and higher pH values, indicating a reduced solubility of the respective Eu(III)–HEDP species formed. This coincides with the results from the solubility studies. Additionally, there is a noteworthy gap in the spectral series for pH values between 7.5 and 9.5. This is due to a pH-shift to lower values during equilibration. Even with multiple pH corrections over several days it was not possible to maintain a constant pH. The reason of this behavior is unclear. One possible explanation is the very slow displacement of a proton upon Eu(III) binding to a partly deprotonated HEDP species such as H_2L^{2-} or HL^{3-}. Raising the pH during adjustment shifts the chemical equilibrium in favor of the complex species. This, in turn, causes more ligand molecules to be deprotonated whereupon the proton liberation results in a decreased pH. A similar effect is observed for the complexation of uranyl ions with PBTC, a polycarboxylate phosphonate ligand, at a lower pH-range (personal communication A. Wollenberg and J. Kretzschmar, manuscript in preparation). PBTC was shown to form strong

intramolecular hydrogen bonds that strongly influence acidity and structure [55]. HEDP's *vicinal* phosphonate groups feature comparably strong intramolecular hydrogen bonds, most likely slowing down Eu(III) complexation and retarding proton abstraction accompanied with a pH drift to lower values.

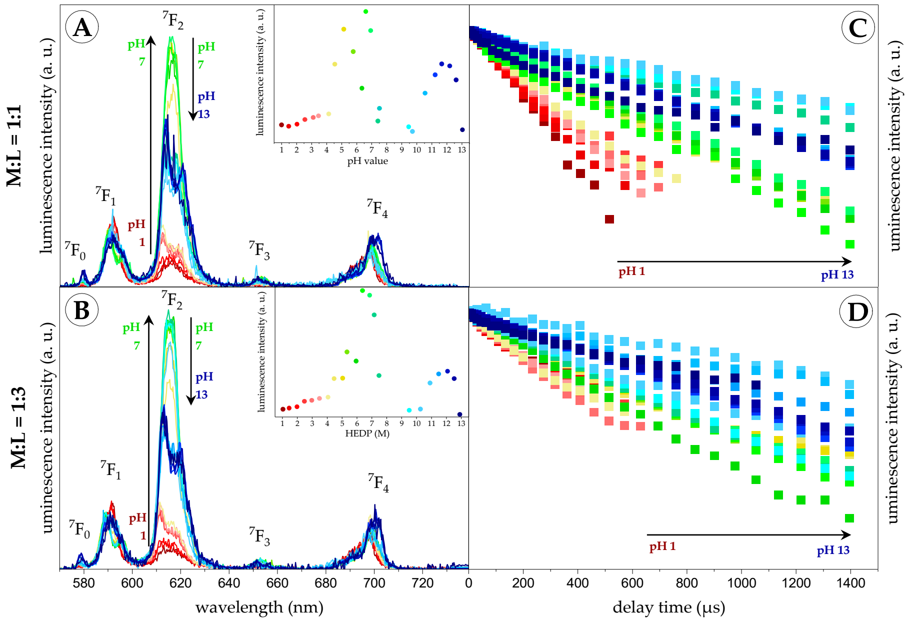

Figure S13: Steady-state luminescence spectra (A and B) and luminescence decay curves (C and D) of **10^{-6} M** u(III) at M:L of 1:1 (A and C) and 1:3 (B and D), without background electrolyte and at (25 ± 1) °C in dependence on the pH (steady-state spectra are normalized to the $^7F_1$ band, and decay curves are normalized to the luminescence intensity at t = 0). Inserts show the pH-dependent raw luminescence intensity.

TRLF spectra and results from the pH series at 10^{-5} M Eu(III) and equimolar M:L are comparable to those at 10^{-6} M. However, at M:L = 1:3, *complex 4* clearly dominates the Eu(III) speciation over a wide pH range (pH 3 – 8) thereby suppressing Eu(III) hydrolysis species as well as HEDP *complexes 3* and *5*.

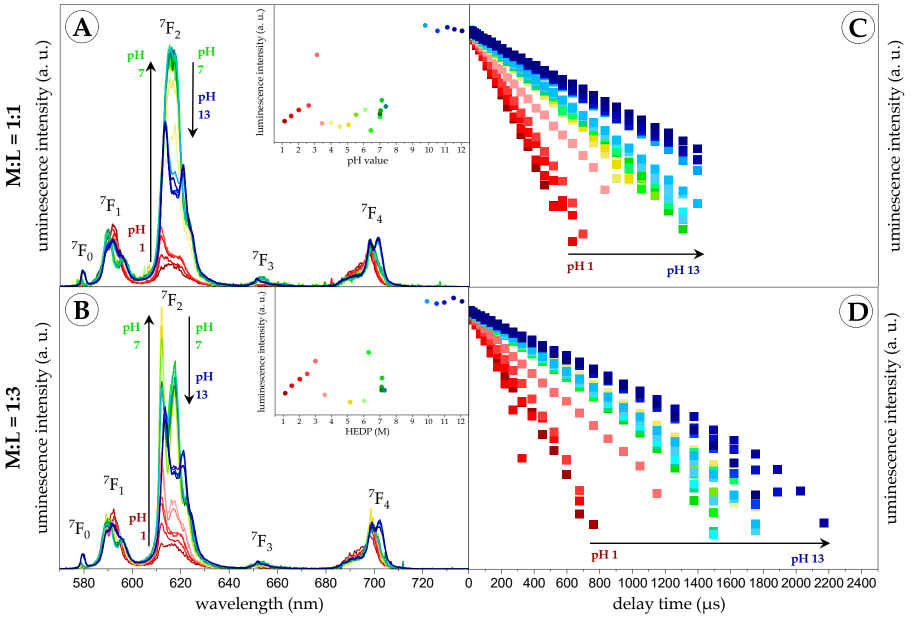

Figure S14: Steady-state luminescence spectra (A and B) and luminescence decay curves (C and D) of the soluble fraction (supernatants) of **10^{-5} M** Eu(III) at M:L of 1:1 (A and C) and 1:3 (B and D), without background electrolyte and at (25 ± 1) °C in dependence on the pH (steady-state spectra are normalized to the $^7F_1$ band area, and decay curves are normalized to the luminescence intensity at t = 0). Inserts show the pH-dependent raw luminescence intensity.

The luminescence spectroscopic parameters of all complexes characterized are given in Table S6.

Table S6: Luminescence spectroscopic properties of the Eu(III) species determined in this study.

	λ (nm)[1]							
**species**	$^7F_0$	$^7F_1$	$^7F_2$	$^7F_3$	$^7F_4$	$R_{E/M}$[2]	τ (µs)[3]	n_{H2O}[4]
Eu^{3+} aqua ion						0.5 - 0.7	110 ± 5	9.0
complex 1	578	589/594	612/620	651	691*/699	1.1 - 1.3	146 ± 4	6.8
complex 2	578	588*/591/594*	612/617	651	691*/699	1.8 - 2.0	209 ± 6	4.8
complex 3	579	590/596	617/623*	653	690*/699	4.5 - 4.7	240 ± 20	3.9
complex 4	579	588/591/597	613/616/624	652	694*/700	4.9 - 5.2	430 ± 5	2.0
complex 5	579	591/595*	614/621	652	690*/699/702	3.4 - 3.6	407 ± 10	2.1

[1] Wavelength of luminescence in the specified ground states with a SD of ± 0.1 nm. Main luminescence bands are given, shoulders are marked with *.

[2] Intensity ratio of the $^7F_2$ band over the $^7F_1$ band with a SD of ± 0.2.

[3] Luminescence lifetime.

[4] Number of water molecules calculated according to Kimura et al. [48] with a SD of ± 0.5.

DFT calculations of solution structures of individual europium(III)–HEDP complexes

All structure models taken into account for DFT are provided in Figure S15.

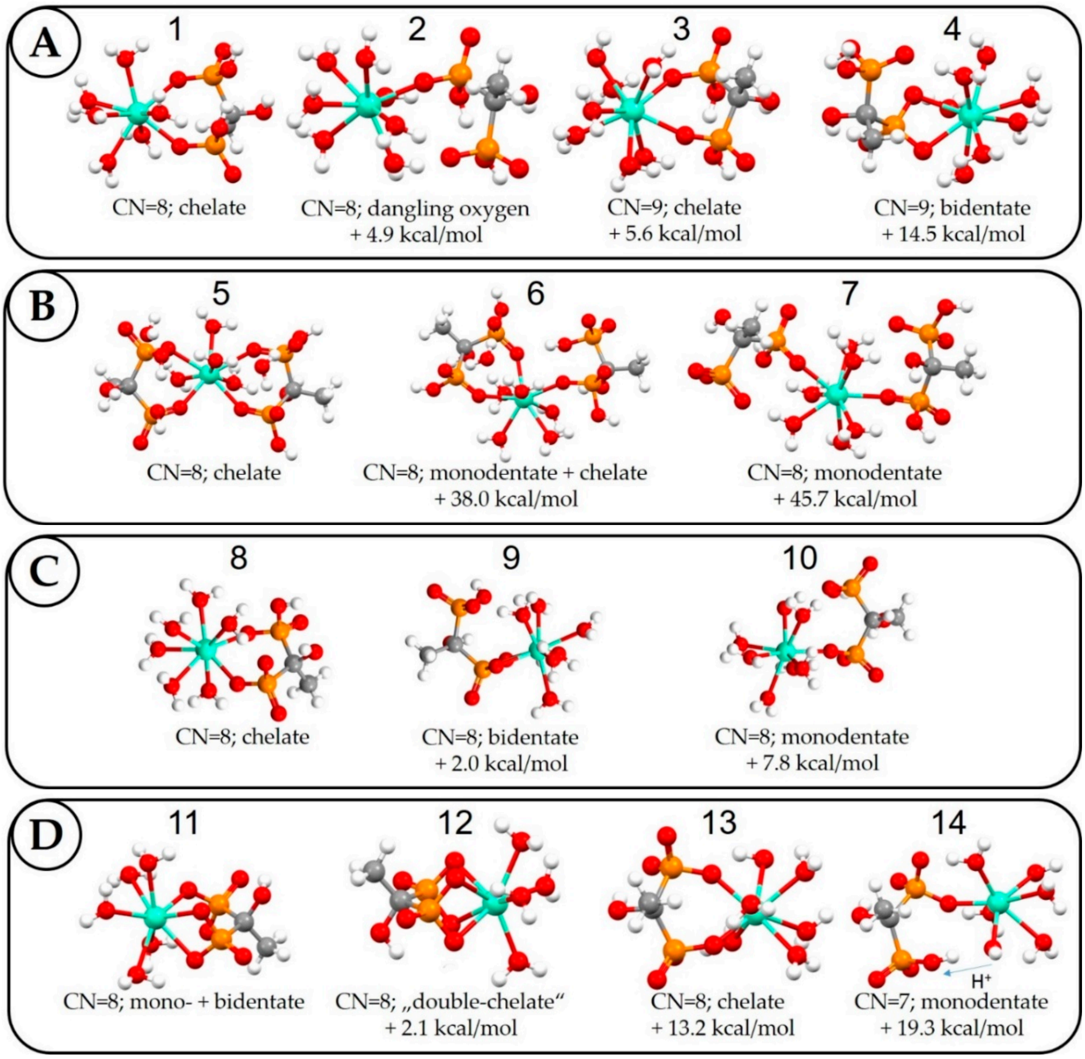

Figure S15: DFT-calculated structure models taken into account: (A) EuH_2L^+ as *complex 1*, (B) $Eu(H_2L)^{2-}$ as *complex 2*, (C) $EuHL^0{}_s$ as *complex 3*, and (D) EuL^- as *complex 5* along with their respective coordination number (CN), binding mode, and energy difference compared to the most stable complex species (always given on the left; white = H, red = O, orange = P, mint = Eu).

Determination of complex formation constants using PARAFAC

To calculate the formation constant of each Eu(III)–HEDP complex all luminescence spectra from HEDP concentration and pH-titration series have been evaluated. In the case of *complexes 1* and *2* (acidic pH), neither solubility nor hydrolysis issues were detected. Hence, log β values were calculated from TRLF spectra of HEDP concentration series measured at pH 1 and 2. In the case of *complex 3* (near-neutral pH), its limited solubility has to be taken into account by using a phase separation algorithm. To assure constant metal and ligand concentrations, the formation constant for this species was determined from pH-titration series for M:L of both 1:1 and 1:3. Since (*i*) *complex 4* is not formed at 10^{-6} M Eu(III), (*ii*) at 10^{-5} M Eu(III), both hardly soluble Eu(III)–HEDP species co-exist and/or mutually interconvert, and (*iii*) the solution structure of this species is yet unknown the calculation of a formation constant is not feasible for this complex. Finally, in the case of *complex 5* (alkaline pH), Eu(III) hydrolysis has to be taken into account when calculating its formation constant from TRLF spectra of HEDP concentration series at pH 11.5. In principle, this would be feasible. However, reference values for log β of $Eu(OH)_3$ and $Eu(OH)_4^-$ in thermodynamic data bases like ANDRA, EQ3/6, HYDRA, NAGRA, CHESS, or Thermochimie differ by 3 – 4 logarithmic units (see Figure S16). Furthermore, these two Eu(III) hydrolysis species are non-luminescent, hence, rendering calculations complicated. Consequently, the formation constant for *complex 5* was determined from pH-titration series with M:L of both 1:1 and 1:3 where Eu(III) hydrolysis plays no role up to pH 13.

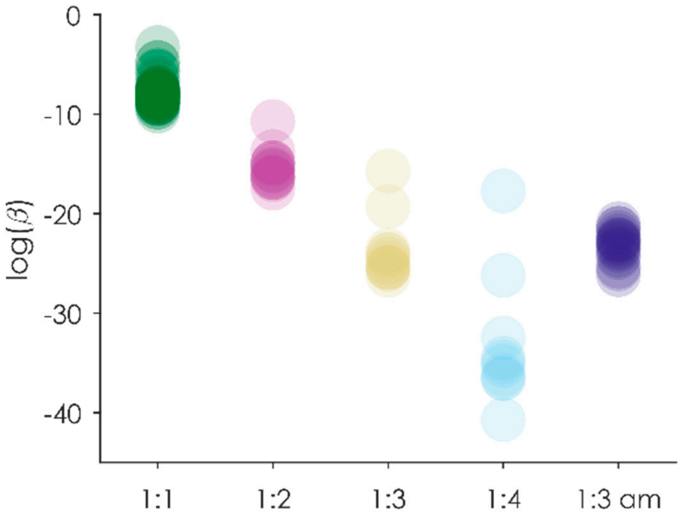

Figure S16: Overview of hydrolysis constants for several $Eu(OH)_n^{3-n}$ (n = 1, 2, 3, 4) species reported in literature [75-86] (x-axis labeling = Eu : OH^-, am = amorphous).

Europium(III) complex formation with HEDP in cell culture medium (detailed)

Results of all ICP-MS measurements of Eu(III) with HEDP in cell culture medium are provided in Figure S17. The order of adding the single constituents is denoted as follows: EH = Eu(III) + HEDP mixed first, then added to the medium, (ME) = first adding Eu(III) to the medium, then HEDP, and (MH) = first adding HEDP to the medium, then Eu(III).

Comparison of the TRLF spectra of Eu(III) and HEDP in cell culture medium with those of Eu–HEDP *complex 4* and the Eu(III)–protein complex formed in cell culture medium is given in Figure S18.

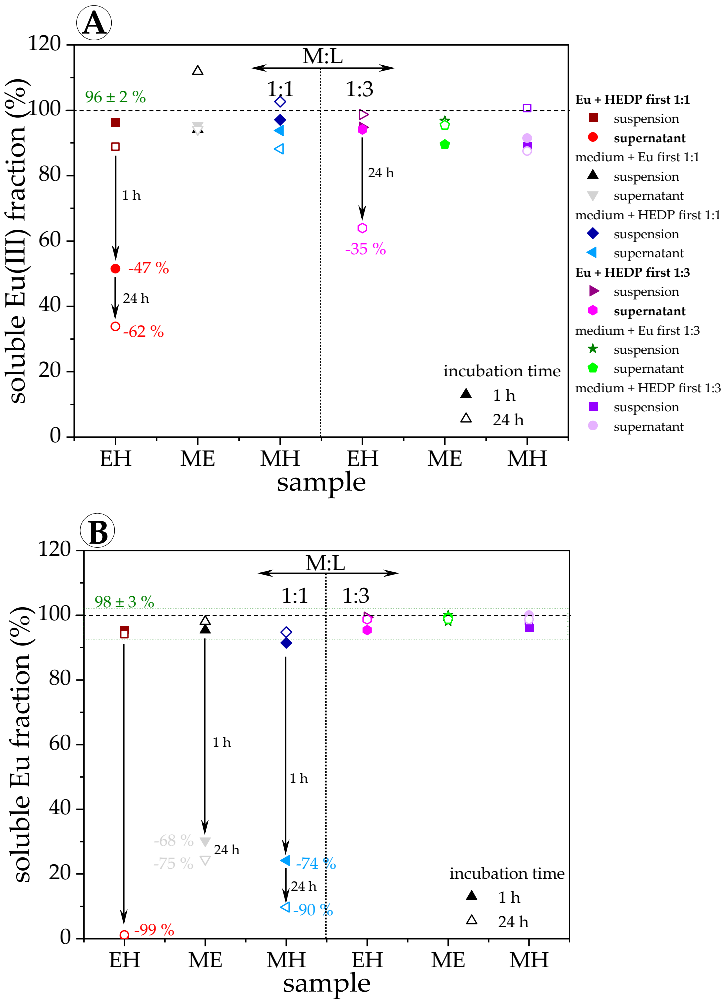

Figure S17: Soluble fraction in the Eu(III)–HEDP cell culture medium system as determined by ICP-MS in suspensions and supernatants at 10^{-5} M (A) and 10^{-3} M Eu(III) (B) at (37 ± 1) °C in dependence on the M:L, the order of constituents' application (EH = Eu + HEDP mixed first, ME = medium + Eu first, MH = medium + HEDP first), and incubation time. Filled and empty symbols correspond to 1 and 24 h of incubation time, respectively.

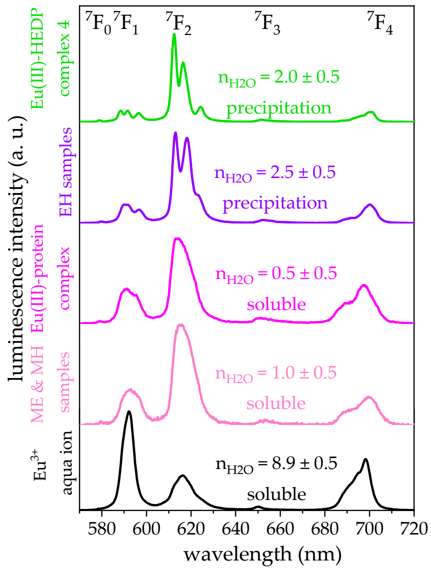

Figure S18: Eu(III) luminescence spectra in EH as well as ME and MH samples in comparison with reference spectra of Eu(III)–HEDP *complex 4* and Eu(III)–protein complex (taken from [52, 53]), along with the number of Eu(III) coordinating water molecules obtained from luminescence decay curves.
